# Formate-driven catalysis and mechanism of an iridium–copper complex for selective aerobic oxidation of aromatic olefins in water†

**DOI:** 10.1039/d0sc06634f

**Published:** 2021-03-16

**Authors:** Yoshihiro Shimoyama, Yasutaka Kitagawa, Yuji Ohgomori, Yoshihiro Kon, Dachao Hong

**Affiliations:** Interdisciplinary Research Center for Catalytic Chemistry, National Institute of Advanced Industrial Science and Technology (AIST) 1-1-1 Higashi Tsukuba Ibaraki 305-8565 Japan hong-d@aist.go.jp; Department of Materials Engineering Science, Graduate School of Engineering Science, Osaka University 1-3 Machikaneyama-cho Toyonaka Osaka 560-8531 Japan; Global Zero Emission Research Center, National Institute of Advanced Industrial Science and Technology (AIST) 1-1-1 Higashi Tsukuba Ibaraki 305-8565 Japan

## Abstract

A hetero-dinuclear Ir^III^–Cu^II^ complex with two adjacent sites was employed as a catalyst for the aerobic oxidation of aromatic olefins driven by formate in water. An Ir^III^–H intermediate, generated through formate dehydrogenation, was revealed to activate terminal aromatic olefins to afford an Ir-alkyl species, and the process was promoted by a hydrophobic [Ir^III^–H]-[substrate aromatic ring] interaction in water. The Ir-alkyl species subsequently reacted with dioxygen to yield corresponding methyl ketones and was promoted by the presence of the Cu^II^ moiety under acidic conditions. The Ir^III^–Cu^II^ complex exhibited cooperative catalysis in the selective aerobic oxidation of olefins to corresponding methyl ketones, as evidenced by no reactivities observed from the corresponding mononuclear Ir^III^ and Cu^II^ complexes, as the individual components of the Ir^III^–Cu^II^ complex. The reaction mechanism afforded by the Ir^III^–Cu^II^ complex in the aerobic oxidation was disclosed by a combination of spectroscopic detection of reaction intermediates, kinetic analysis, and theoretical calculations.

## Abstract 2

A hetero-dinuclear Ir^III^–Cu^II^ complex with two adjacent sites was employed as a catalyst for the aerobic oxidation of aromatic olefins driven by formate and promoted by a hydrophobic interaction in water.

## Graphical abstract

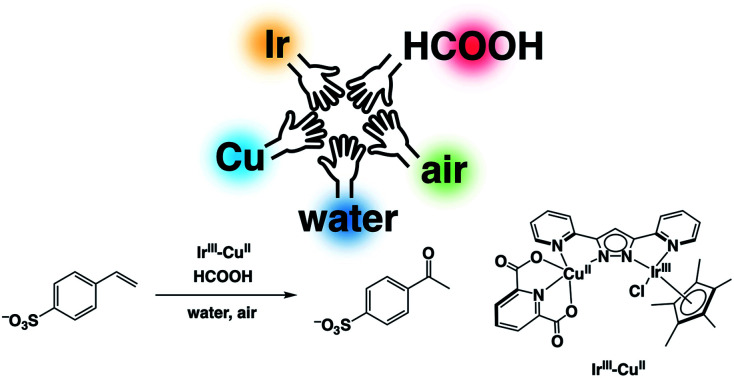

## Introduction

Molecular oxygen (O_2_) is an ideal oxidant for the catalytic oxidation of organic substrates as water is generated in the reactions without producing any waste products.^1–3^ Constructing a system that enables regio/stereoselective or partial oxidations of substrates by O_2_ for fine chemical and pharmaceutical productions is particularly desirable.^4,5^ In nature, regioselective oxidations are accomplished by cytochrome P450,^6–8^ MMO (methane monooxygenase),^9,10^ and DβM (dopamine β-monooxygenase)^11,12^ that function as catalysts to monohydroxylate steroid derivatives, methane, and dopamine under air, respectively. These enzymes accomplish such selective oxidations by promoting interactions between the organic substrates and the enzyme active site which often includes hydrophobic cavities or non-covalent interaction centers.^13–18^

Inspired by these enzymes, artificial catalysts such as metal complexes have been utilized for selective and partial oxidations of organic substrates.^19–23^ Biomimetic metal complexes were, in fact, reported to possess a hydrophobic environment around their reaction sites capable of suppressing over-oxidations. In addition, water used as a reaction solvent can promote hydrophobic interactions between organic substrates and reactive intermediates of metal complexes in the reaction centres, playing a critical role in enhancing reactivity.^24–26^

For the aerobic oxidations performed in aqueous solutions, Wacker-type catalysts have been widely studied in basic and applied synthesis over the past decades.^27,28^ The Wacker-type systems are often composed of a palladium catalyst and an electron mediator such as copper(ii) ions,^27^ polyoxometalates,^29^ or quinone derivatives.^30^ Metal–hydride (M–H) species have been receiving increasing attention in the Wacker-type oxidations because, by using these species, in contrast to the palladium case, the catalysis exhibits unique behaviour and proceeds without the need of an additional electron mediator.^31,32^ This approach, however, is limited by the difficulty of generating M–H species by reactive hydride sources, which are reductants not usually adopted in an aqueous solutions for oxidation reactions. Thus, a method that allows us to generate M–H species in aqueous solution and that allows us to build a hydrophobic environment suitable for selective oxidations of organic substrates would be highly desirable.

The formation of a M–H species in aqueous solutions has been previously reported by using a mild hydride source such as formic acid.^33–36^ To the best of our knowledge, there have been no reports on selective aerobic oxidation of olefins to methyl ketones in water catalysed by M–H species that can exhibit a hydrophobic interaction with substrates. Mechanistic insights into selective aerobic oxidations performed by M–H species in water have yet to be gained. Herein, we report that an Ir^III^–Cu^II^ hetero-dinuclear complex^37^ successfully catalyses Wacker-type oxidation of aromatic olefins driven by formate to produce corresponding methyl ketones in water. We found that a hydrophobic interaction between an Ir–H intermediate and the substrate aromatic ring in water promoted the selective aerobic oxidation of aromatic olefins. We have revealed that the iridium centre worked as the organometallic activation site of formate and olefins, while the copper centre serves as the electronic modulating site by improving the overall reactivity toward dioxygen under acidic conditions. We discuss the mechanistic insights into the catalytic Wacker-type oxidation of water-soluble styrene derivatives performed by the Ir^III^–Cu^II^ complex based on spectroscopic detection of reaction intermediates, kinetic analysis, and theoretical calculations.

## Results and discussion

**Fig. 1:**
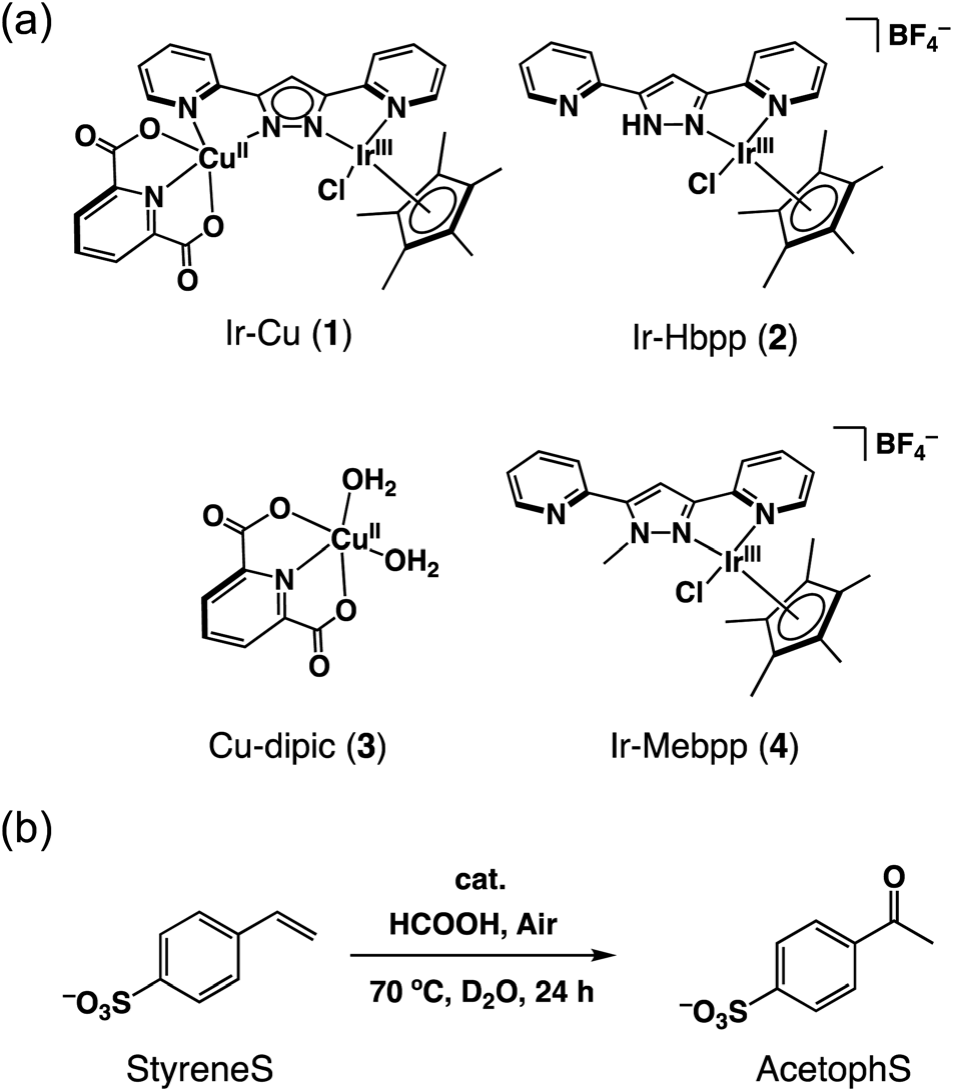
(a) Catalysts **1–4** used in this work. (b) Selective aerobic oxidation of *p*-styrenesulfonate (StyreneS) to acetophenonesulfonate (AcetophS) in water.

The Ir^III^–Cu^II^ complex (**1**) and reference catalysts, [(Cp*)Ir^III^Cl(Hbpp)](BF_4_) (Ir-Hbpp, **2**), [Cu^II^(dipic)(OH_2_)_2_] (Cu-dipic, **3**) and [(Cp*)Ir^III^Cl(Mebpp)](BF_4_) (Ir-Mebpp, **4**) as shown in Fig. 1a, were synthesized and characterized according to literature procedures.^37^ The aerobic oxidation reactions afforded by **1–4** were conducted using *p*-styrenesulfonate (StyreneS) as a water-soluble model substrate (Fig. 1b). Acetophenonesulfonate (AcetophS) as the corresponding methyl ketones obtained from the Wacker-type oxidation of StyreneS was quantified by ^1^H NMR spectroscopic analysis using DSS (sodium 4,4-dimethyl-4-silapentanesulfonate) as an internal standard (Fig. S1†). The selectivity for methyl-ketone reached to almost 100% and only a negligible formation of aldehyde was observed from the reaction on the basis of the ^1^H NMR spectral analysis. The results for the selective aerobic oxidation of StyreneS to AcetophS in formate buffer are summarized in Table 1. The use of **1** as a catalyst successfully affords AcetophS with yield and TON values of 11 and 112, respectively (Table 1, entry 1). Control experiments were conducted to investigate the cooperative catalysis of **1** using the mononuclear metal complexes (**2–4**), which constitute the moiety of **1**, as catalysts. The Ir^III^ and Cu^II^ complexes used as mononuclear catalysts, however, did not show any reactivities towards StyreneS oxidation (Table 1, entries 2–5). The results highlight the exceptional catalysis of **1** obtained for the selective aerobic oxidation of StyreneS to AcetophS.

**Table 1:** Yields and TONs obtained from the catalytic aerobic oxidation of StyreneS in aqueous solutions^a^

Entry	Cat.	AcetophS	
		Yield, %	TON
1	Ir–Cu (**1**)	11	112
2	Ir-Hbpp (**2**)	n.d.	n/a
3	Cu-dipic (**3**) + Ir-Mebpp (**4**) (1 : 1)	n.d.	n/a
4	Ir-Mebpp (**4**)	n.d.	n/a
5	Cu-dipic (**3**)	n.d.	n/a
6	—	n.d.	n/a
7	Ir–Cu (**1**)^b^	n.d.	n/a
8	Ir–Cu (**1**)^c^	n.d.	n/a
9	Ir–Cu (**1**)^d^	26	263
10	Ir–Cu (**1**)^e^	23	232
11	Ir–Cu (**1**)^f^	58	580
12	Cu-dipic (**3**) + Ir-Mebpp (**4**)^f^	n.d.	n/a
13	Ir–Cu (**1**)^g^	n.d.	n/a

a Conditions: [cat.] = 0.10 mM, [HCOOH] = 0.20 M, [StyreneS] = 0.10 M, solvent: D_2_O (1.0 mL), reaction temp.: 70 °C. Reaction time: 24 h. Under air. Yield (%) = 100 × ([product]/[StyreneS]), TON = [product]/[cat.].

b Under Ar.

c Without HCOOH.

d With excess of **3** (5.0 mM).

e With excess of **3** (10 mM).

f With excess of **3** (5.0 mM) in formate buffer (0.60 M, [HCOOH] : [HOCONa] = 3 : 1).

g With excess of **3** (5.0 mM) in DMA/D_2_O (5 : 1, v/v) (0.60 M formate buffer, [HCOOH] : [HOCONa] = 3 : 1).

In addition, no reactivity was observed in the absence of a catalyst and formic acid or under an Ar atmosphere (entries 6–8), showing that both oxygen and formic acid are needed for the aerobic oxidation of StyreneS to generate AcetophS. As previously reported,^37^ hetero-dinuclear metal complexes, Ir^III^–Co^II^ and Ir^III^–Ni^II^, were also employed in the oxidation reactions but did not catalyse the Wacker-type oxidation of StyreneS to AcetophS (Table S1†). This result is ascribed to be the higher H_2_ evolution rates obtained by using Ir–Ni and Ir–Co complex as compared to that of the Ir–Cu complex shown in our previous report.^37^ Additionally, another possible explanation can be that the Co^II^ and Ni^II^ centres of Ir^III^–Co^II^ and Ir^III^–Ni^II^ complexes are allowed from their sixth coordination to form an octahedral geometry that may interfere with the access of StyreneS as compared to a pseudo-square-pyramidal geometry of the Cu^II^ center in the Ir^III^–Cu^II^ complex. These results demonstrate that the copper centre in **1** plays a significant role in providing a specific reaction environment suitable for the selective aerobic oxidation of StyreneS to AcetophS.

As evidenced by ESI-TOF-MS measurements (Fig. S2†), complex **1** was found to dissociate to complexes **2** and **3** during the oxidation reactions, resulting in its deactivation. The dissociation may go through hydrolysis of **1** with an equilibrium between **1** and the corresponding mononuclear Ir and Cu complexes under high proton concentrations at 70 °C. The addition of an extra amount of complex **3** (5.0–10 mM) into the reaction solutions containing **1** significantly enhanced both the reaction yields and TONs (Table 1, entries 9 and 10). The presence of an excess of **3** in the reaction mixtures may, thus, shift the equilibrium towards **1** maintaining the structure of the active catalyst to afford the high catalytic activity observed here for the aerobic oxidation of StyreneS. The reaction conditions were, then, optimized by altering the formic acid/sodium formate concentration ratios as well as their absolute concentrations. The highest TONs for **1** were obtained at the 3 : 1 (mol mol^−1^) [HCOOH] : [HCOONa] ratio (Fig. S3a†) and at a formate concentration of 0.60 mM (Fig. S3b†). Using the optimized conditions, the yield of AcetophS and the reaction TON for **1** reached 58% and 580, respectively, in the selective aerobic oxidation of StyreneS (Table 1, entry 11). Additionally, no products were obtained from the reaction solution of **4** with excess of **3** under the same optimized conditions (Table 1, entry 12), suggesting the crucial role played by the presence of Cu(ii) adjacent to Ir(iii) to afford the aerobic oxidation. Furthermore, the use of a less polar solvent, *N*,*N*-dimethylacetamide (DMA)/D_2_O (5 : 1, v/v), resulted in no production of AcetophS in the Wacker-type oxidation, even under the optimized conditions (Table 1, entry 13). This result suggests that the use of water may promote a hydrophobic interaction between **1** and StyreneS to enable the selective aerobic oxidation shown here.

**Fig. 2:**
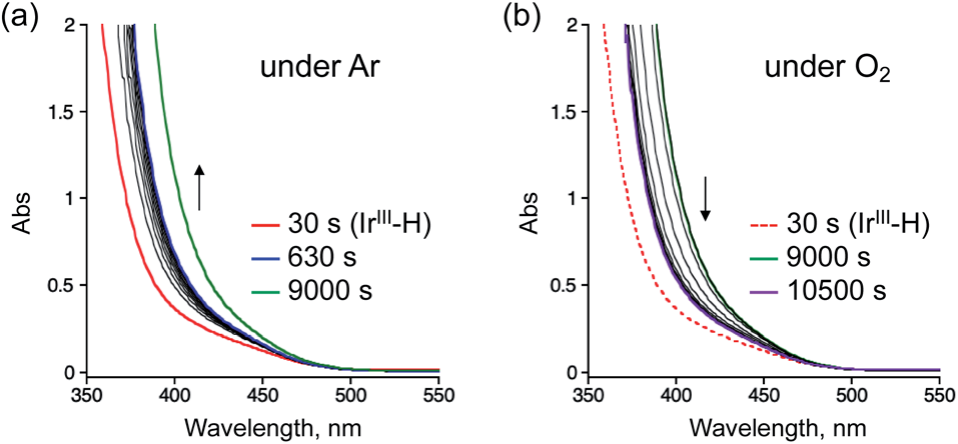
UV-vis absorption spectral changes of **1** (0.10 mM) with **3** (5.0 mM) in formate buffer (0.60 M, [HCOOH] : [HCOONa] = 3 : 1) in the presence of 0.10 M StyreneS (a) under Ar (0–9000 s) and (b) under O_2_ (9000–10500 s) at 70 °C. O_2_ gas was introduced into the solution at 9000 s.

To reveal the reaction mechanism for the selective aerobic oxidation of StyreneS afforded by **1**, we measured UV-vis absorption spectral changes of **1** in the presence/absence of StyreneS, as shown in Fig. 2 and S4.† The absorption band with a shoulder at 380 nm, which can be assigned to the Ir^III^–H species in **1** (**Ir(H)-Cu**),^37^ was observed in formate buffer under Ar in the absence of StyreneS (Fig. S4a†). The absorption band showed almost no changes by introducing O_2_ into the solution without StyreneS (Fig. S4b†). The result suggests that no reactions were taking place between **Ir(H)-Cu** and O_2_. On the other hand, the further absorption growth at 405 nm was observed after formation of **Ir(H)-Cu** in the presence of StyreneS under Ar (Fig. 2a). When Ar was replaced with O_2_, the absorption band immediately decreased and saturated within 1500 seconds (Fig. 2b). These results indicate that **Ir(H)-Cu** in formate buffer selectively reacted with StyreneS followed by reaction with molecular oxygen. The excess of **3** added did not interfere with the aerobic oxidation reaction although its absorption appeared around 780 nm due to the d–d transitions of **3** (Fig. S5†). Additionally, the continuous absorption growth at 405 nm was not observed in the absence of excess of **3** (Fig. S6†), probably due to the dissociation of the Cu-dipic moiety from **1** as evidenced by Fig. S2.† The UV-vis absorption spectral changes observed here are consistent with the catalytic results that the addition of an excess of **3** improves the yield of AcetophS due to preservation of the **Ir(H)-Cu** active structure.

**Scheme 1:**
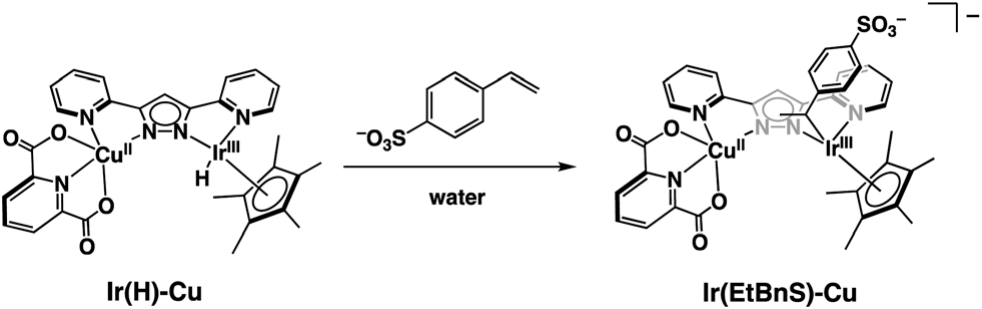
Schematic representation of **Ir(EtBnS)-Cu** formation from the reaction between **Ir(H)-Cu** and StyreneS in water.

Based on the UV-vis spectral changes of **1** observed in formate buffer under Ar, the **Ir(H)-Cu** can be assumed to react with StyreneS to form an Ir-alkyl complex, [(Cp*)Ir(C_8_H_8_SO_3_)(bpp)Cu(dipic)]^−^ (**Ir(EtBnS)-Cu**) (Scheme 1). Olefin insertion into the M–H bond of mononuclear metal complexes has been previously reported to form iridium-alkyl species.^38–41^ The formation of **Ir(EtBnS)-Cu** was supported by our electron-spray ionization mass spectrometry (ESI-MS) measurements in which a peak cluster at *m*/*z* = 1006.96, assigned to be [[(Cp*)Ir(C_8_H_8_SO_3_)(bpp)Cu(dipic)]^−^ + HCOOH]^−^ (calcd. for [M]^−^: *m*/*z* = 1007.12), was observed (Fig. S7a†). The peak cluster was not obtained in the absence of StyreneS under Ar (Fig. S7b†). We have attempted to detect **Ir(EtBnS)-Cu** by ^1^H NMR spectroscopy; however, the paramagnetic nature of the Cu^II^ center did not allow for a satisfactory peak assignment. When using DMA/H_2_O (5 : 1, v/v) as a solvent, the absorption growth at 405 nm derived from the formation of **Ir(EtBnS)-Cu** was not observed (Fig. S8†). Considering that the Wacker-type oxidation did not proceed in DMA/H_2_O solvent (Table 1, entry 13), the results clearly indicate that **Ir(EtBnS)-Cu** is formed only in net water, which is a key finding of this work.

Since metal-hydroperoxide (M–OOH) species have so far been proposed as intermediates in olefin oxidations to produce corresponding ketones using late-transition metal catalysts, we have employed H_2_O_2_ as an oxidant instead of O_2_ and HCOOH to examine the selective oxidation of StyreneS by **1**.^42–45^ Remarkably, AcetophS was not produced in the oxidation of StyreneS when using H_2_O_2_ in the place of O_2_ and HCOOH (Table S2†). Therefore, we conclude that the oxidation of StyreneS performed by **1** involved the formation of **Ir(EtBnS)-Cu** generated by the reaction between **Ir(H)-Cu** and StyreneS through olefin insertion into an Ir^III^–H bond instead of an Ir–OOH species.

**Fig. 3:**
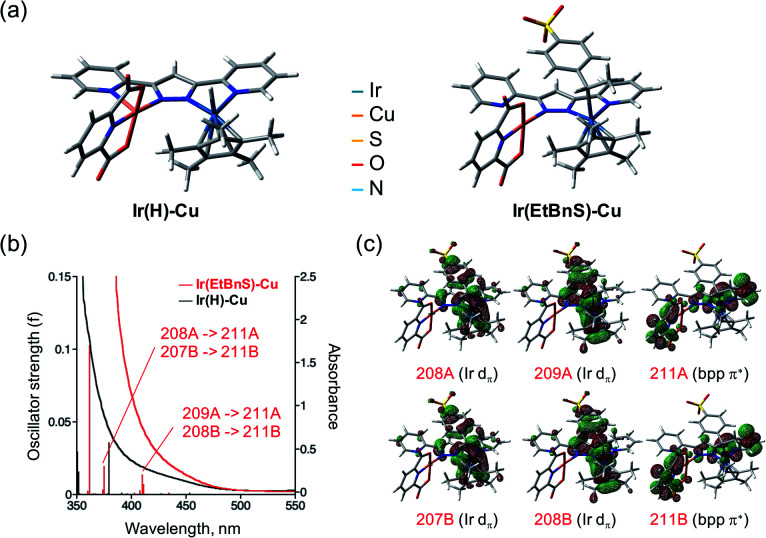
(a) Optimized structures of **Ir(H)-Cu** (left) and **Ir(EtBnS)-Cu** (right) as the reaction intermediates calculated at the UB3LYP-D3BJ/LanL2TZ(f) (Ir), 6-31G* (Cu, H, C, O, N), 6-31+G* (S) level of theory. (b) UV-vis absorption spectra of **Ir(H)-Cu** (black) and **Ir(EtBnS)-Cu** (red) formed at 30 s and 9000 s respectively after addition of **1** under Ar overlaid with TD-DFT calculations on the optimized structures of **Ir(H)-Cu** (black trace) and **Ir(EtBnS)-Cu** (red trace). (c) Contributing molecular orbitals of **Ir(EtBnS)-Cu** (208A, 209A, 211A, 207B, 208B, and 211B) to the absorption at 410.22 nm and 375.14 nm obtained from the structural optimization.

In order to further support the formation of **Ir(EtBnS)-Cu**, we conducted theoretical calculations on the optimized structures of **Ir(EtBnS)-Cu** (Fig. 3). The calculations showed a clear π–π interaction occurring between the benzene ring of StyreneS and the bpp ligand (*ca.* 3.1 Å) in **Ir(EtBnS)-Cu** (Fig. 3a).^46^ This hydrophobic π–π interaction corroborates the hypothesis of an important water solvent contribution to the driving force for the formation of Ir-alkyl species. The energy of the hydrophobic π–π interaction of **Ir(EtBnS)-Cu** was roughly estimated to be 32.3 kcal mol^−1^ by the energy difference between single-point calculations at the UB3LYP and UB3LYP-D3BJ levels of theory (see ESI† for details on the computation of this energy). It should be noted that the energy values relative to the π–π interactions can be overestimated by the calculations and, thus, they should be taken into account for a qualitative rather than a quantitative analysis. The results from our TD-DFT calculations also indicate that the oscillator strength of **Ir(EnBnS)-Cu** exhibits good consistency in the region of the observed absorption band at 405 nm, which can be assigned to the metal-to-ligand charge transfer (MLCT) band from dπ orbitals of Ir^III^ (208A, 209A, 207B and 208B) to a π* orbitals of bpp and dipic ligands (211A and 211B) (Fig. 3b and c).

A π–π stacking of aromatic substrates with catalysts as a pre-equilibrium process has been proposed before.^47^ The occurrence of the π–π interaction between substrates and the bpp ligand of **1** was demonstrated experimentally by oxidation of other terminal olefins with/without an aromatic ring including 4-vinylbenzoic acid, 4-vinylpyridine, allylphenylethersulfonate and allylsulfonate (Table 2); only the substrates having a vinyl group next to the aromatic ring were oxidized by **1** to produce the corresponding methyl ketones. Although the allylphenylether derivative can in principle engage in a π–π interaction with **Ir(H)-Cu** as StyreneS, the vinyl group lies too far from the Ir–H bond as a reactive site in this situation. The substrate limitation observed clearly reflects the requirement for a hydrophobic π–π interaction between the substrate aromatic rings and **1** in water, shedding light on the mechanism of this reaction.

**Table 2:** Yields and TONs obtained from the selective aerobic oxidation of aromatic olefins by **1** in water^a^

Substrates	Products	Yield, %	TON
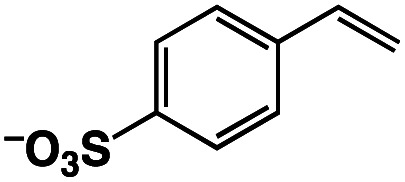	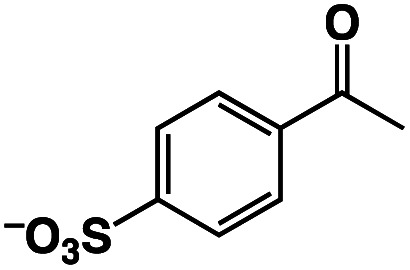	58	580
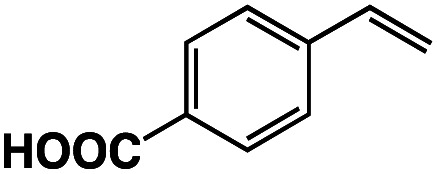	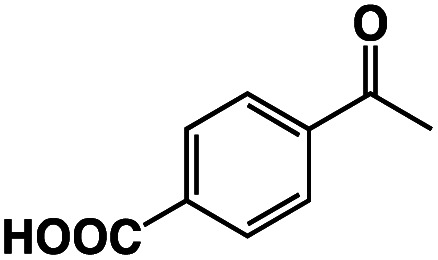	3	33
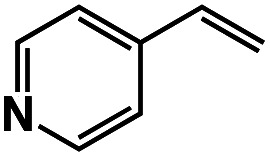	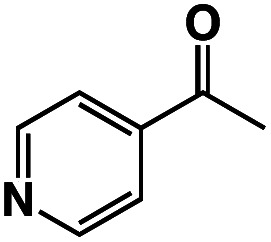	17	169
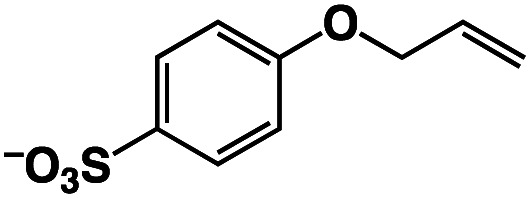	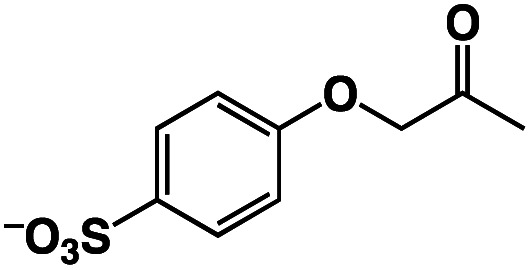	n.d.	n/a
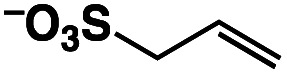	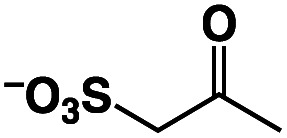	n.d.	n/a

a Conditions: [**1**] = 0.10 mM, [**3**] = 5.0 mM, solvent: 0.60 M formate buffer ([HCOOH] : [HCOONa] = 3 : 1) in D_2_O (1.0 mL), [substrate] = 0.10 M, reaction temp.: 70 °C. Reaction time: 24 h. Under air. Yield (%) = 100 × ([product]/[StyreneS]), TON = [product]/[cat.].

**Fig. 4:**
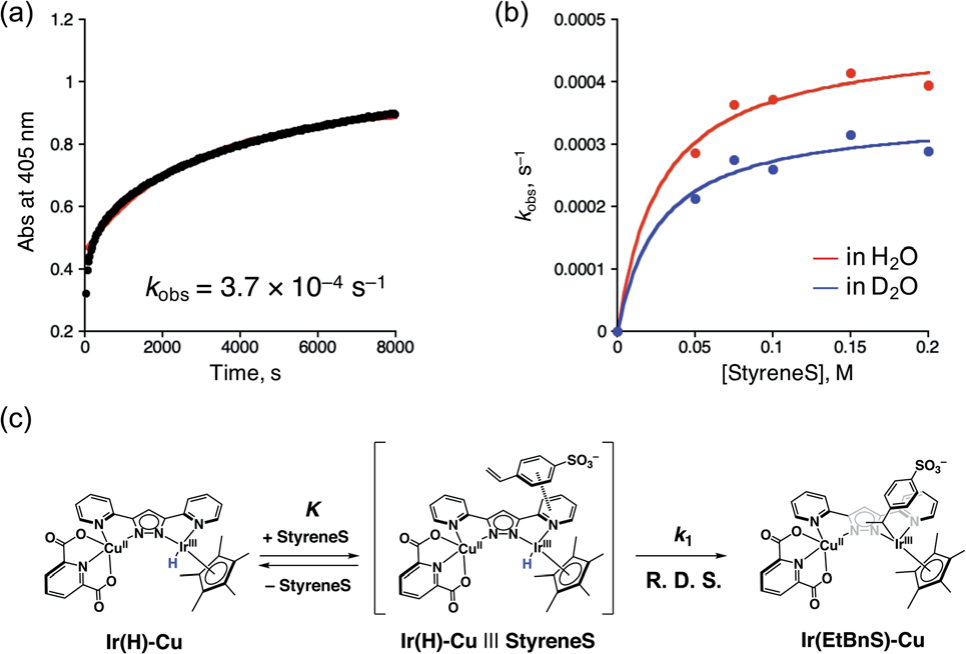
(a) Time-profile of absorbance at 405 nm (black dots) and the fitting curve (red line) obtained from **1** (0.10 mM) with **3** (5.0 mM) in formate buffer (0.60 M, [HCOOH] : [HCOONa] = 3 : 1) in the presence of 0.10 M StyreneS under Ar. (b) The determined *k*^H^_obs_ (red trace) and *k*^D^_obs_ (blue trace) values against StyreneS concentration (0.050–0.20 M) and the fitting curve (red line). (c) Schematic representation of the pre-equilibrium process.

In order to gain more insights into the formation of **Ir(EtBnS)-Cu**, we performed kinetic studies under Ar, as shown in Fig. 4. The absorption spectral changes observed at 405 nm, derived from **Ir(EtBnS)-Cu**, were analysed based on pseudo-first-order kinetics, which exhibited an apparent rate constant (*k*_obs_) of 3.7 × 10^−4^ s^−1^ (Fig. 4a). The *k*_obs_ (s^−1^) obtained was plotted against the StyreneS concentrations and the kinetics exhibited a saturation behaviour (Fig. 4b). Both the first-order rate constant (*k*^H^_1_) and the equilibrium constant (*K*^H^) were determined to be 4.7 × 10^−4^ s^−1^ and 3.6 × 10 M^−1^, respectively, based on a pre-equilibrium between the benzene ring of StyreneS and the bpp ligand (Fig. 4c). The π–π stacked structure of the adduct (**Ir(H)-Cu|||StyreneS**) was obtained from DFT calculations (Fig. S9†) and the stabilization energy derived from the π–π interaction was roughly estimated to be 23.1 kcal mol^−1^. The use of D_2_O as a solvent gave *k*^D^_1_ and *K*^D^ values of 3.5 × 10^−4^ s^−1^ and 3.7 × 10 M^−1^, respectively, and the kinetic isotope effect (KIE, *k*^H^_1_/*k*^D^_1_) was determined to be 1.4. On the other hand, *K*^H^/*K*^D^ was determined to be 1.0, indicating no isotope effect on the pre-equilibrium between the Ir–H species and StyreneS. Considering that the iridium-hydride species undergoes H/D exchange with the solvent as evidenced previously,^37^ the KIE of *k*^H^_1_/*k*^D^_1_ indicates that the olefin insertion into the Ir–H bond is involved in the rate-determining step (R. D. S.).

In comparison to the StyreneS oxidation performed by **1**, complex **4** showed no reactivity with the same substrates despite the formation of the iridium-alkyl complex (**Ir(EtBnS)-Mebpp**), which was observed by ESI-TOF-MS measurements (Fig. S10†). The difference in reactivity between **Ir(EtBnS)-Cu** and **Ir(EtBnS)-Mebpp** was evaluated by conducting electrochemical measurements on **1** and **4** in formate buffer in the presence of StyreneS. The oxidation waves of **Ir(EtBnS)-Cu** and **Ir(EtBnS)-Mebpp** were observed at +0.34 V and +0.82 V (*vs.* Ag/AgCl), respectively (Fig. S11†), indicating that **Ir(EtBnS)-Cu** is readily oxidized compared to **Ir(EtBnS)-Mebpp**. Metal-alkyl species with a lower ionization potential (*i.e.* negative redox potential) have been reported to exhibit fast O_2_ insertion into a metal–carbon bond without changing metal valency.^48^ Thus, the negative redox potential of **Ir(EtBnS)-Cu** explains its higher reactivity toward O_2_ insertion, as compared to that of **Ir(EtBnS)-Mebpp**, without involving a redox event of the Ir(iii/iv) center.

As the bpp ligand bears a formal negative charge in complex **1** while it is neutral in complex **4**, the charge difference could offer a possible explanation for the negative shift in redox potentials observed between the π–π interacting complexes **Ir(EtBnS)-Cu** and **Ir(EtBnS)-Mebpp**. To understand the role of the Cu(ii) center and the difference in electronic properties, we have postulated the **Ir(EtBnS)-bpp** species by removing the Cu(ii)-dipic moiety from **Ir(EtBnS)-Cu** and conducted DFT calculations on **Ir(EtBnS)-Cu** and **Ir(EtBnS)-bpp** (both have the same charge) as shown in Fig. S12 and Table S3.† Virtually no difference in the Mülliken charges have been found on both the ligands and the iridium center (Table S3†). It is notable that the O_2_ insertion into iridium-alkyl complexes occurs only when copper(ii) is present in the moiety. Although the Ir-bpp complex, which is the deprotonated form of **2**, cannot form under the acidic catalytic conditions, it is stabilized by the presence of the Cu(ii) moiety in **1**. The Cu(ii) center would help to modulate the electronic properties of the **Ir(EtBnS)-Cu** intermediate, allowing a more favorable O_2_ insertion in contrast to **Ir(EtBnS)-Mebpp** by lowering the redox potential. It should be noted that our DFT calculations were carried out only for the olefin insertion step to form **Ir(EtBnS)-Cu**.; thus, a possible influence of electronic effects derived from Cu(ii) in other steps of the catalytic cycle cannot be ruled out.

**Scheme 2:**
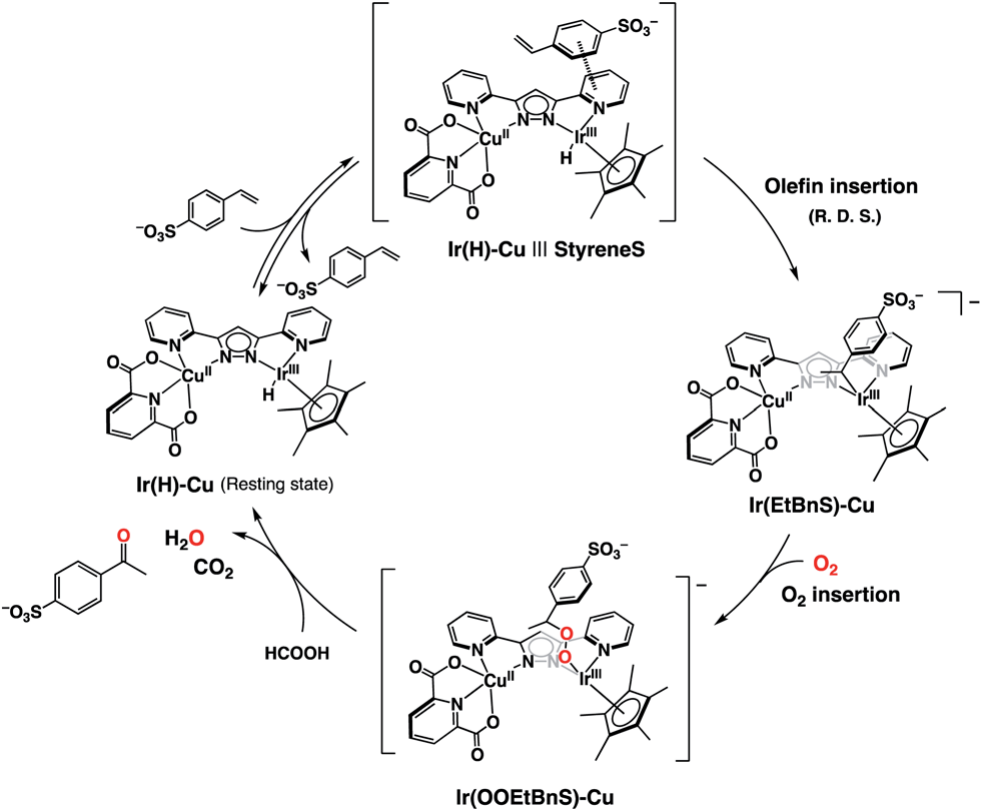
Proposed catalytic cycle for the selective aerobic oxidation of StyreneS by **1**.

On the basis of the combined experimental and theoretical evidence, the catalytic cycle of the dioxygen-coupled oxidation of terminal olefins performed by **1** is proposed as shown in Scheme 2. First of all, the Ir–hydride complex (**Ir(H)-Cu**), generated by the reaction of **1** and formate, forms an adduct (**Ir(H)-Cu|||StyreneS**) due to a π–π interaction between the substrate aromatic rings and the bpp ligand, in a pre-equilibrium process. An iridium-alkyl complex (**Ir(EtBnS)-Cu**) is then formed by the insertion of the terminal olefin in the Ir–H bond as the R. D. S. of the catalytic cycle. Molecular oxygen inserts into the Ir^III^-alkyl bond to produce a putative iridium-alkylperoxo complex (**Ir(OOEtBnS)-Cu**). Finally, the heterolysis of the O–O bond gives the corresponding methyl ketone and the initial **Ir(H)-Cu** complex is regenerated from formic acid. The metal-alkylperoxido species formed by the O_2_ insertion into the metal-alkyl bond has been previously reported^49,50^ and was also proposed as a reaction intermediate in Wacker-type oxidations of olefins.^31,32^ The DFT-optimized structure and the electronic properties of **Ir(OOEtBnS)-Cu** are shown in Fig. S13 and Table S4.† According to the TD-DFT calculations performed on **Ir(OOEtBnS)-Cu**, a characteristic oscillator strength was observed at 434.47 nm. On the other hand, no absorption growth around 434 nm was experimentally observed in the reaction of **Ir(EtBnS)-Cu** and O_2_ due to the fast cleavage of an O–O bond in **Ir(OOEtBnS)-Cu** to produce AcetophS.^51^ In addition, **Ir(EtBnS)-Cu** was not observed in the UV-vis spectral changes of the reaction solution under air, but **Ir(H)-Cu** (Fig. S14†). The results are consistent with the observation that the R. D. S. of the catalytic cycle under air is the olefin insertion into **Ir(H)-Cu**.

## Conclusions

In summary, we have demonstrated the exceptional catalytic activity of **1** in the selective aerobic oxidation of StyreneS to AcetophS as compared to that of the corresponding individual mononuclear Ir and Cu complexes derived from **1** and the mixture of these complexes in water. The formation of iridium-alkyl species from the StyreneS insertion into the iridium-hydride species in **1** was the key step (R. D. S.) of the Wacker-type oxidation of aromatic olefins in water observed here, where a hydrophobic π–π interaction between the aromatic ring of olefins and the bpp ligand of **1** engages in a pre-equilibrium process, based on evidence from DFT calculations and kinetic analysis. Only olefin substrates with an alkene group located next to the aromatic ring were selectively oxidised into corresponding methyl ketones, which highlights the significance of the occurrence of hydrophobic π–π interactions between substrates and **1** in water. Electrochemical measurements performed on the iridium-alkyl species indicate that the Cu moiety in **1** enhances the reactivity of the iridium-alkyl species toward O_2_ in acidic conditions. This work will open the possibility of expanding the use of hetero-dinuclear metal complexes possessing structurally adjacent sites in molecular catalysts that exploit catalyst–substrate hydrophobic interactions for efficient and selective oxidation reactions in water.

## Experimental

### General

^1^H NMR spectral measurements were performed on a JEOL JNM-ECX 400 spectrometer using 4,4-dimethyl-4-silapentanesulfonic acid sodium salt (DSS) as an internal standard. UV-vis spectral measurements at 70 °C were performed using SCINCO UV-visible spectrophotometer S-3100 equipped with a cryostat (CoolSpeK UV USP-203, UNISOKU Co., Ltd.). LC-MS measurements were performed on a SHIMADZU LCMS-2020 spectrometer. Purified water (18.2 MΩ cm) was obtained from a Milli-Q system (Direct-Q3 UV, Millipore). Electrochemical measurements were carried out using HZ-7000 HOKUTO DENKO.

### Chemicals

Ir–Cu (**1**),^37^ Ir-Hbpp (**2**),^37^ Cu-dipic (**3**),^52^ Ir-Mebpp (**4**),^37^ Ir–Co,^37^ and Ir–Ni,^37^ complexes were synthesized according to the literature procedures. Sodium *p*-styrenesulfonate (StyreneS), sodium formate, sodium 4-acetylbenzenesulfonate, sodium allylsulfonate, and 4-vinylbenzoic acid were purchased from TCI Chemical Co. Ltd. Formic acid and 4-vinylpyridine were purchased from Fujifilm Wako Co. D_2_O was purchased from ISOWATER. All chemicals were used without further purification.

### General procedure for catalytic oxidations of olefins

An olefin substrate and DSS (internal standard) were dissolved into a formate buffer (D_2_O). A certain amount (10–30 μL) of Ir–Cu (**1**) in 2,2,2-trifluoroethanol solution was added to the substrate solution, and then the reaction mixture was stirred at 70 °C to start the reaction. After each reaction time, the reaction mixture was analysed with ^1^H NMR spectroscopy to probe for products generation.

### Determination of *k*_obs_, *k*_1_, and *K* values for the reaction between **1** and StyreneS under Ar

The UV-vis absorption changes at 405 nm under Ar were analysed based on pseudo-first kinetics to obtain apparent rate constants (*k*_obs_) by the curve-fitting based on eqn (1). The *k*_obs_ values obtained were plotted against StyreneS concentration using eqn (2) to determine the first-order rate constant (*k*_1_) and the equilibrium constant.

1 Abs = Abs_0_ + ΔAbs (1 − exp(−*k*_obs_/*t*))

2 *k*_obs_ = *k*_1_*K*[StyreneS]/(1 + *K*[StyreneS]).

### DFT calculations

DFT calculations were carried out for the geometry optimization and frequency calculations using the unrestricted B3LYP-D3BJ functional to consider van der Waals interactions.^53–55^ We used the basis sets of LanL2TZ(f)^56^ for Ir, 6-31G*^57,58^ for Cu, H, C, N, and O atoms, and 6-31+G^57,58^ for the S atom. The total spin state of the complex including the Cu atom was fixed to be a doublet. The optimized geometries were confirmed that they did not have any imaginary frequencies. TD-DFT calculations^59^ were performed for 30 excited states on the basis of the optimized structures. All the calculations were performed in water solvent using a polarizable continuum model (PCM) adopting the integral equation formalism variant (IEFPCM).^60^ The Gaussian 09 (Revision D.01) program package^61^ was used for all DFT calculations.

## Author contributions

Y. O. synthesized and characterized the catalysts. Y. S. conducted most of the catalytic tests, intermediate detection, and kinetic analysis. Y. Kitakawa performed the DFT calculations. Y. S., Y. Kon, and D. H. conceived the ideas and designed the work. All authors contributed to the discussion on the mechanism. Y. S. and D. H. wrote the draft with data from all authors, and all authors edited and reviewed the draft. D. H. supervised all aspects of the work. All authors approved the final version of the draft.

## Conflicts of interest

There are no conflicts to declare.

## Supplementary Material

SC-012-D0SC06634F-s001
