# Supplementary material for: Formate-driven catalysis and mechanism of an iridium–copper complex for selective aerobic oxidation of aromatic olefins in water
Source: Chem Sci. 2021 Mar 16;12(16):5796–803. doi: 10.1039/d0sc06634f (PMC8179673; doi:10.1039/d0sc06634f)
Supplement: SC-012-D0SC06634F-s001 [file SC-012-D0SC06634F-s001.pdf]

Electronic Supplementary Material (ESI) for Chemical Science.
This journal is © The Royal Society of Chemistry 2021

Electronic Supplementary Information

Formate-Driven Catalysis and Mechanism of an Iridium-Copper Complex for Selective Aerobic Oxidation of Aromatic Olefins in Water

Yoshihiro Shimoyama,[a] Yasutaka Kitagawa,[b] Yuji Ohgomori,[a] Yoshihiro Kon,[a,c] and Dachao Hong,*[,a,c]

[a] Interdisciplinary Research Center for Catalytic Chemistry, ‡Global Zero Emission Research Center, National Institute of Advanced Industrial Science and Technology (AIST), 1-1-1 Higashi, Tsukuba, Ibaraki 305-8565, Japan

[b] Department of Materials Engineering Science, Graduate School of Engineering Science, Osaka University, 1-3 Machikaneyama-cho, Toyonaka, Osaka 560-8531, Japan

[c] Global Zero Emission Research Center, National Institute of Advanced Industrial Science and Technology (AIST), 1-1-1 Higashi, Tsukuba, Ibaraki 305-8565, Japan.

* To whom correspondence should be addressed.
E-mail: hong-d@aist.go.jp (D. H.)

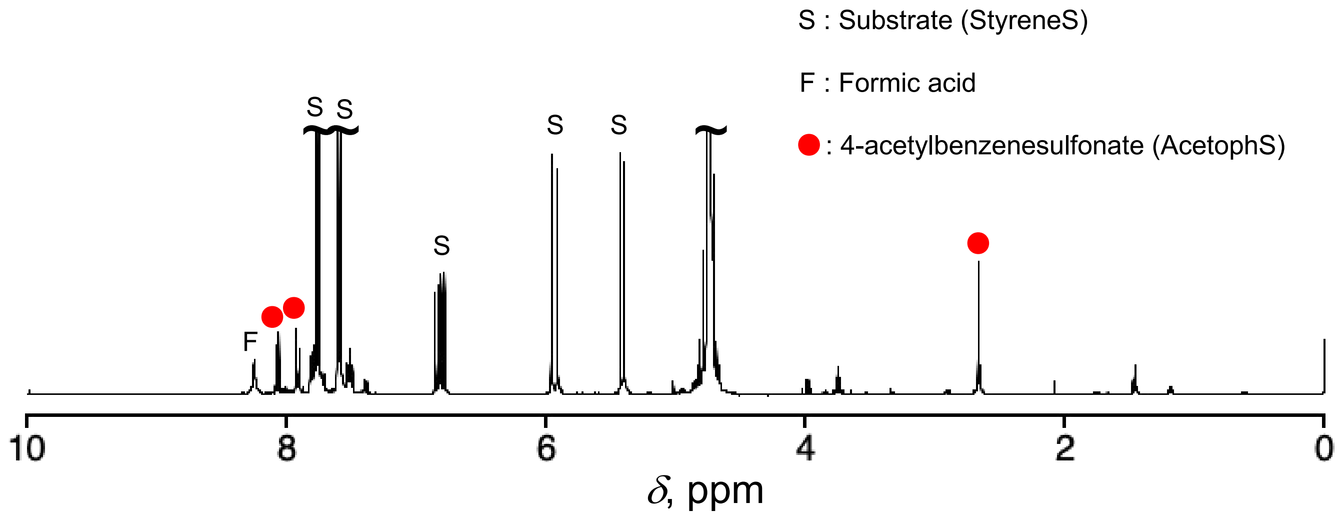

Fig. S1 ^{1}H NMR spectrum of the reaction mixture after StyreneS oxidation performed by **1** in D_2O under air. Conditions: [**1**]: 0.10 mM, solvent: 0.20 M HCOOH in D_2O, reaction temp.: 70 ºC, reaction time: 24 h.

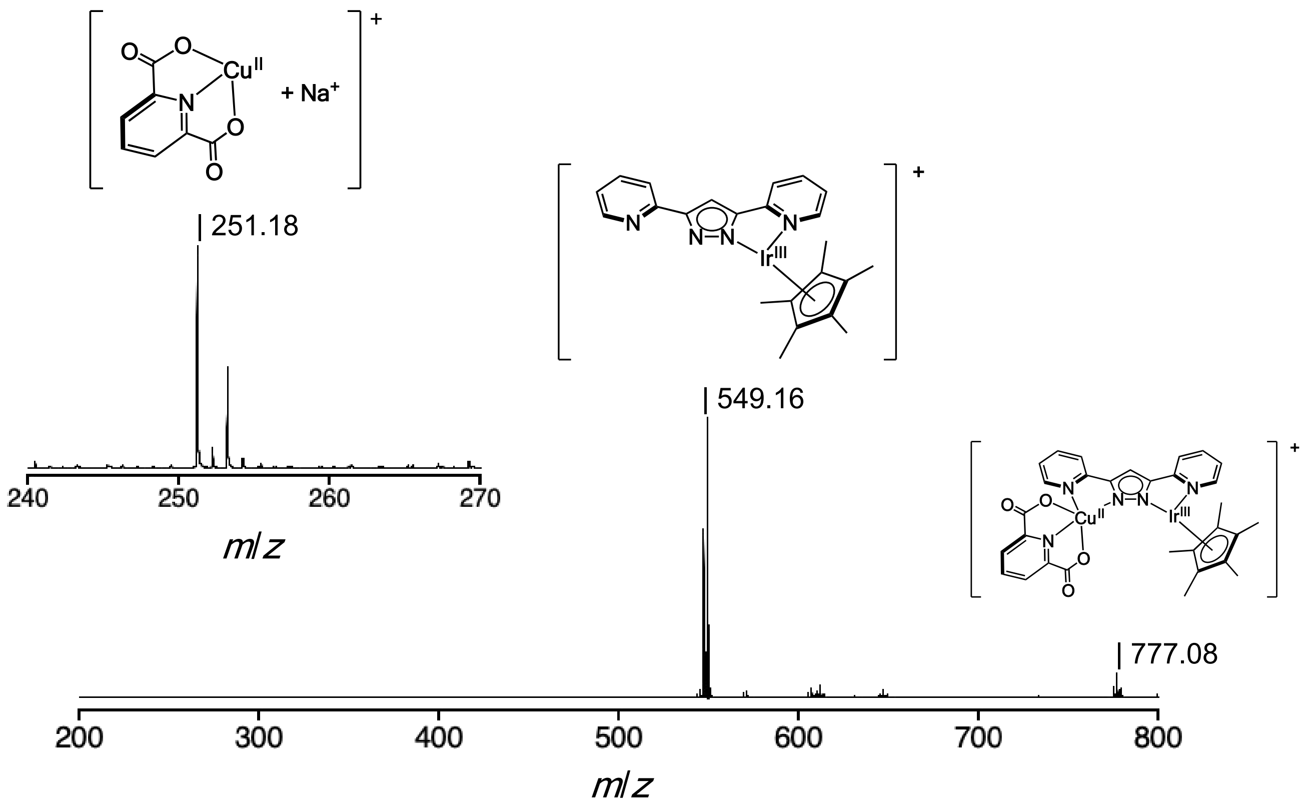

Fig. S2 ESI-TOF-MS spectrum of Ir-Cu (**1**) in formic acid (0.20 M) aqueous solution. The sample was diluted with MeOH for the measurement. The inset shows a MS peak of the Cu-dipic fragment observed by adjusting the voltage parameter during the measurements.

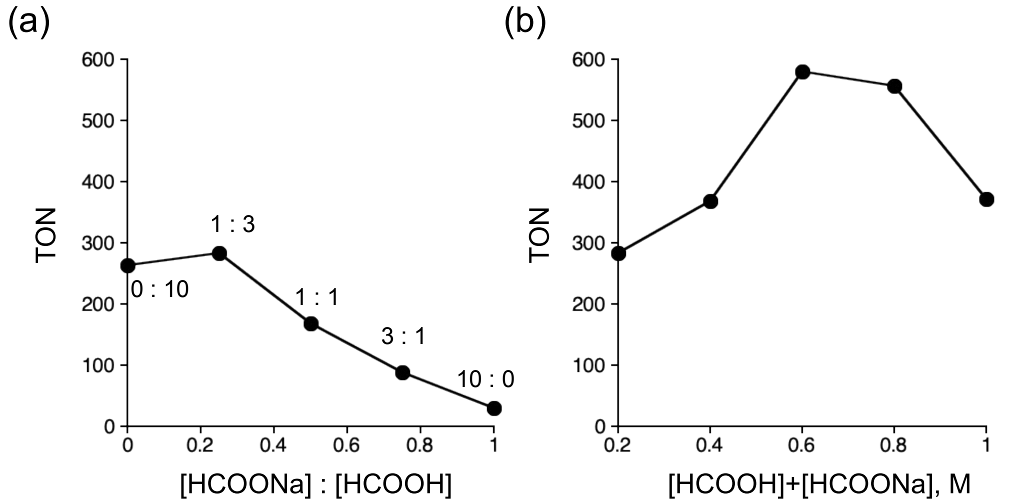

Fig. S3 Optimization of the reaction conditions through change of formic acid or sodium formate concentrations. Change of the formic acid/sodium formate ratio (a) and of the total concentration of the formate buffer ([HCOOH]:[HCOONa] = 3:1) (b). Reaction conditions: [**1**] = 0.10 mM, [**3**] = 5.0 mM, [StyreneS] = 0.10 M, solvent: Formate buffer in D_2O. Reaction temp.: 70 ºC. Reaction time: 24 h. Under air.

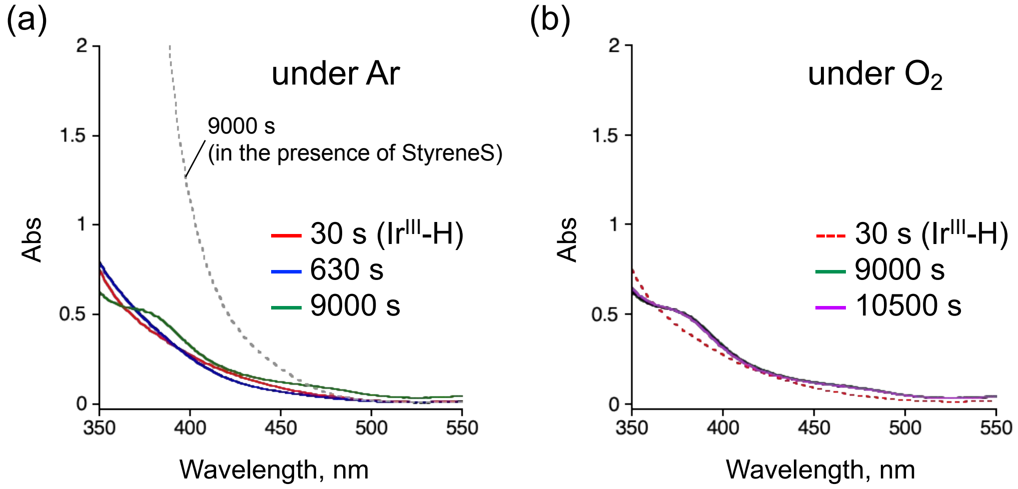

Fig. S4 UV-Vis absorption spectral changes of **1** (0.10 mM) with **3** (5.0 mM) in formate buffer (0.60 M, [HCOOH]:[HCOONa] = 3:1) in the absence of StyreneS (a) under Ar (0–9000 s), and (b) under O_2 (9000–10500 s) at 70 ºC. O_2 gas was introduced into the solution at 9000 s. The slight absorption changes at 380 nm and 450–500 nm may be derived from the partial decomposition of the **Ir(H)-Cu** complex.

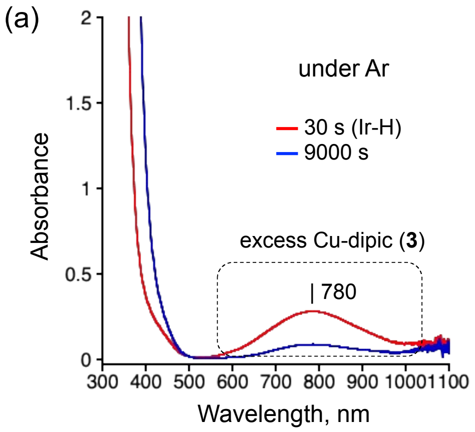

Fig. S5 UV-Vis absorption spectral changes of **1** (0.10 mM) and **3** (5.0 mM) in formate buffer (0.60 M, [HCOOH]:[HCOONa] = 3:1) in the presence of 0.10 M StyreneS (a) under Ar (0–9000 s), and (b) under O_2 (9000–10500 s) at 70 ºC. O_2 gas was introduced into the solution at 9000 s. The broad absorption band at 780 nm due to the excess of **3** (*d*-*d* transition band, black-dotted square) partially decreased in the presence of StyreneS and recovered under O_2 probably due to redox reactions between **3** and Cu^{I} or/and Cu^{II} species.

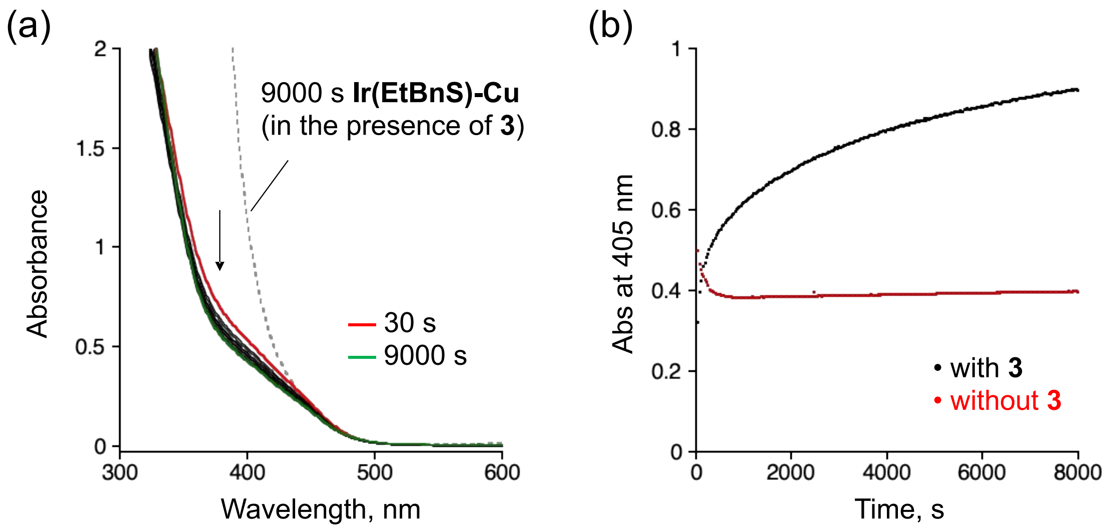

Fig. S6 (a) UV-Vis absorption spectral changes of **1** (0.10 mM) without **3** in formate buffer (0.60 M, [HCOOH]:[HCOONa] = 3:1) in the presence of 0.10 M StyreneS under Ar (0–9000 s) at 70 ºC. (b) The time profiles of the absorption changes monitored at 405 nm in the presence (black) or absence (red) of **3**.

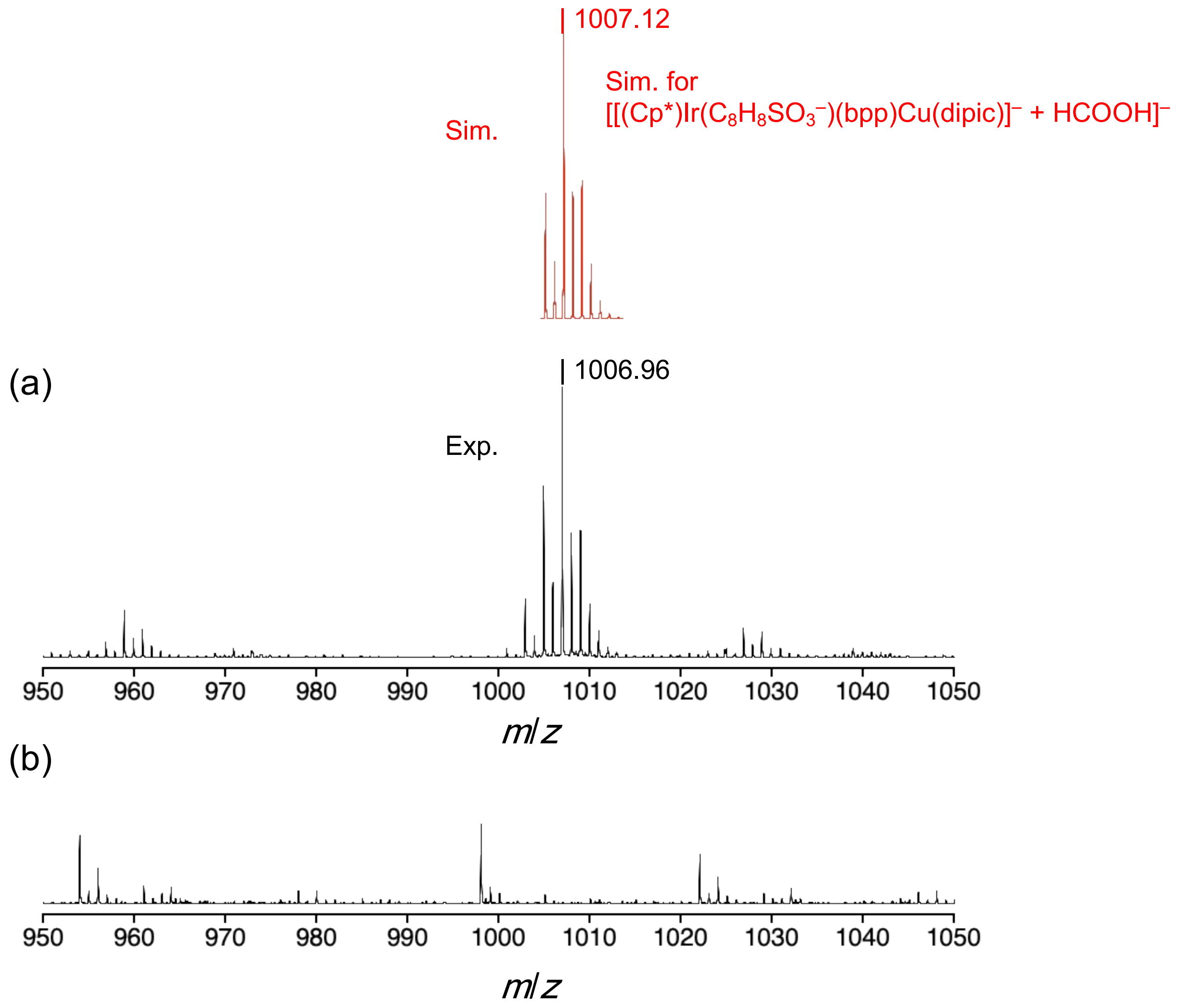

Fig. S7 (a) ESI-TOF-MS spectra of **Ir(EtBnS)-Cu** in MeOH (black) and the simulation spectrum (red). The sample solution was prepared by diluting the reaction mixture with MeOH obtained from the reaction of **1** in formate buffer in the presence of StyreneS under Ar. Reaction conditions: [**1**] = 0.10 mM, [**3**] = 5.0 mM, [StyreneS] = 0.10 M, solvent: formate buffer (0.60 M, [HCOOH]:[HCOONa] = 3:1) in H_2O, reaction temp.: 70 ºC. (b) ESI-TOF-MS spectra of the reaction solution without StyreneS under Ar. Capillary exit voltage: –100 V, Skimmar voltage: –50 V.

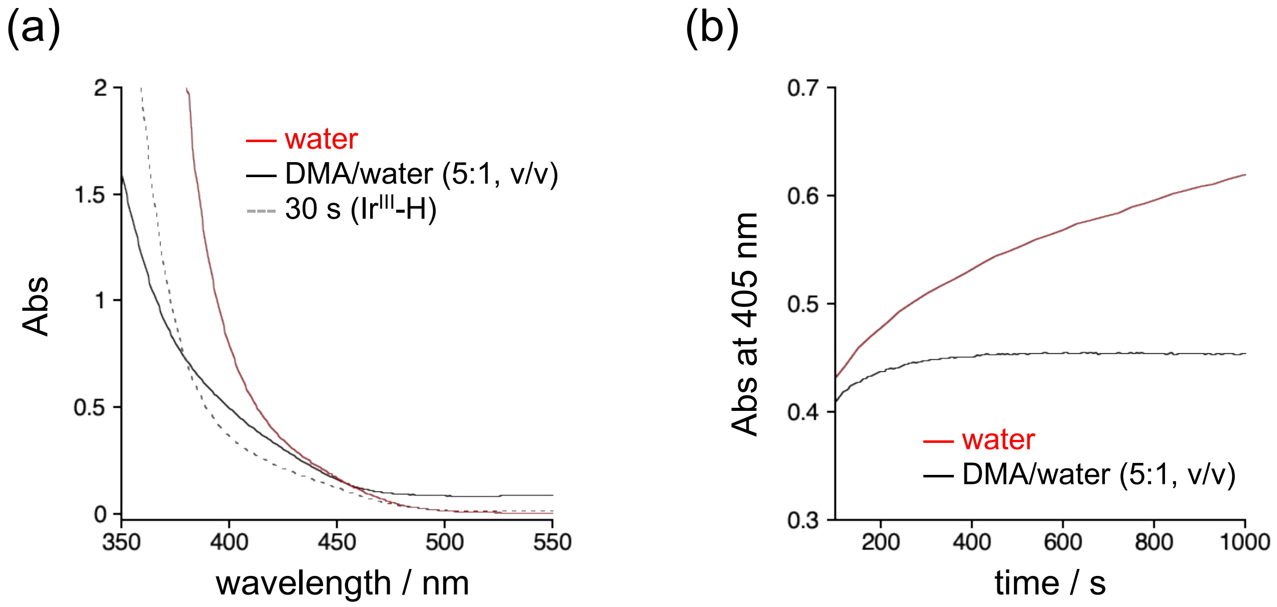

Fig. S8 (a) UV-Vis absorption spectra of **1** in water (red line), DMA/water (5:1, v/v, black line) at 9000 s, and Ir-H species observed at 30 s in water (gray dashed line) in the presence of 0.60 M formic acid/formate and 0.10 M StyreneS under Ar. (b) Time-profile of the absorbance of **1** monitored at 405 nm in water (red) and DMA/water (5:1, v/v, black). Reaction conditions: [**1**] = 0.10 mM, [**3**] = 5.0 mM, solvent: formate solution (0.60 M, [HCOOH]:[HCOONa] = 3:1), reaction temp.: 70 ºC. Under Ar

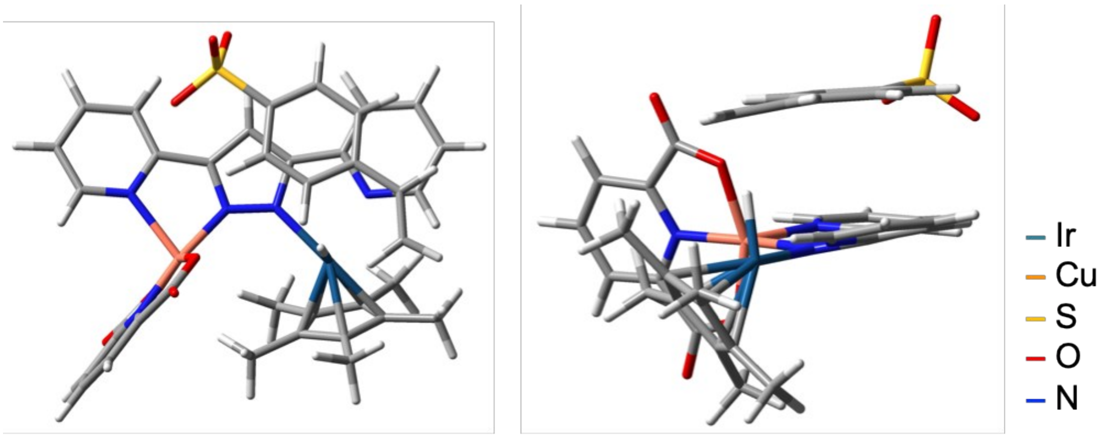

Fig. S9 A DFT-optimized structure of **Ir(H)-Cu|||StyreneS** (top view and side view) calculated at the unrestricted B3LYP-D3BJ/LanL2TZ(f) (Ir), 6-31G* (Cu, H, C, O, N), 6-31+G* (S) level of theory. The strength of the π-π interaction was estimated to be 23.14 kcal/mol by calculating the energies obtained by B3LYP and B3LYP-D3BJ functional sets. The method of energy estimation is described below.

The π-π interaction energy between the **Ir(H)-Cu** and **StyreneS** is calculated based on the equations; $\Delta\Delta E = \Delta E_{\mathrm{B3LYP}} - \Delta E_{\mathrm{B3LYP\text{-}D3BJ}}$, $\Delta E_{\mathrm{X}} = E$(**Ir(H)-Cu|||StyreneS)** – {E(**Ir(H)-Cu**) + E(**StyreneS**)}, where E(**Y**) and ΔE_{X} (X=B3LYP, B3LYP-D3BJ) represent the calculated total energies of the whole and partial structures at the optimized **Ir(H)-Cu|||StyreneS** geometry by B3LYP and B3LYP-D3BJ level of theory, the energy difference of the whole and partial structures calculated by B3LYP and B3LYP-D3BJ level of theory, respectively. Thus, the energy difference ($\Delta\Delta E$) between $\Delta E_{\mathrm{B3LYP\text{-}D3BJ}}$ and $\Delta E_{\mathrm{B3LYP}}$ indicates the π-π interaction energy because $\Delta E_{\mathrm{B3LYP\text{-}D3BJ}}$ includes the π-π interaction energy while $\Delta E_{\mathrm{B3LYP}}$ does not. The calculated total energy of each model is listed as follows.

Functional set	Calculated total energies / Hartree			ΔE_{X} / Hartree
	**Ir(H)-Cu|||StyreneS**	**Ir(H)-Cu**	**StyreneS**	
B3LYP	-4412.93164178	-3479.93039606	-933.00880821	0.00756249
B3LYP-D3BJ	-4413.20937058	-3480.13069114	-933.04937159	-0.02930785

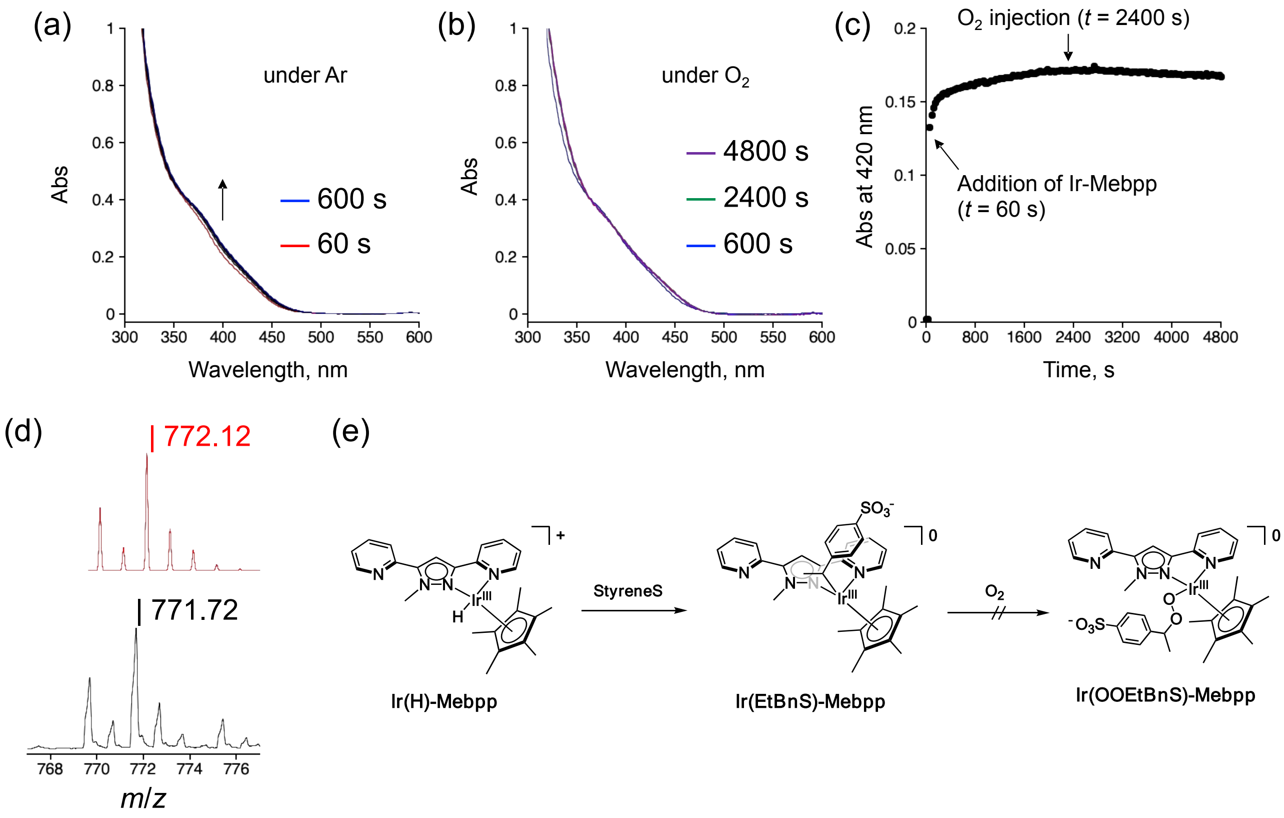

Fig. S10 (a) UV-Vis absorption spectral changes of **4** in formate buffer in the presence of 0.10 M StyreneS under (a) Ar (0–600 s) and (b) O_2 (2400–4800 s). O_2 gas was introduced into the solution at 2400 s. (c) Time profile of the absorbance at 420 nm (black dots). (d) An ESI-TOF-MS spectrum of [**Ir(EtBnS)-Mebpp** + K^+]$^+$ in MeOH-diluted sample of the reaction mixture of **4** with StyreneS in 0.60 M formate buffer (black) and its simulated spectrum (red). (e) Schematic representation of an insertion of StyreneS into the Ir-H bond in Ir(H)-Mebpp. Reaction conditions: [**4**] = 0.10 mM, solvent: formate buffer (0.60 M, [HCOOH]:[HCOONa] = 3:1) in H_2O, reaction temp.: 70 ºC.

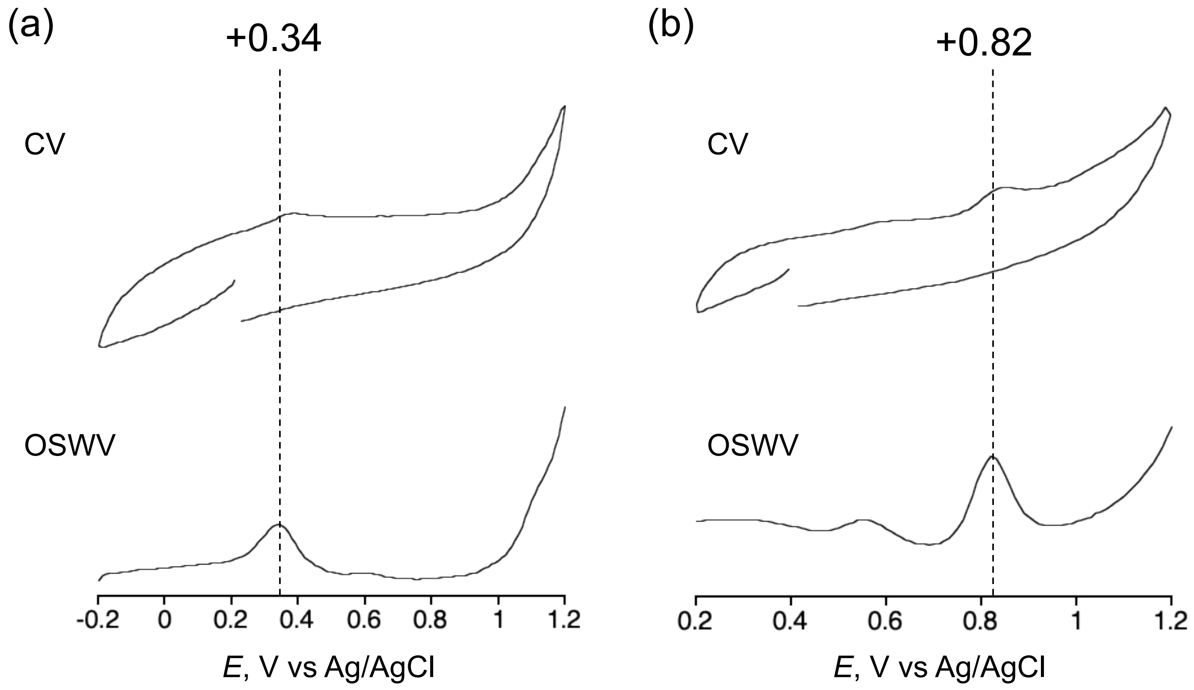

Fig. S11 Cyclic voltammograms (CV, top) and Osteryoung square-wave voltammograms (OSWV, bottom) of **1** (a) and **4** (b) in the presence of 0.10 M StyreneS. Conditions: [**1**] = [**4**] = 0.50 mM, solvent: formate buffer (0.60 M, [HCOOH]:[HCOONa] = 3:1) in H_2O, temp.: room temperature. WE: Glassy carbon, CE: Pt wire, RE: Ag/AgCl.

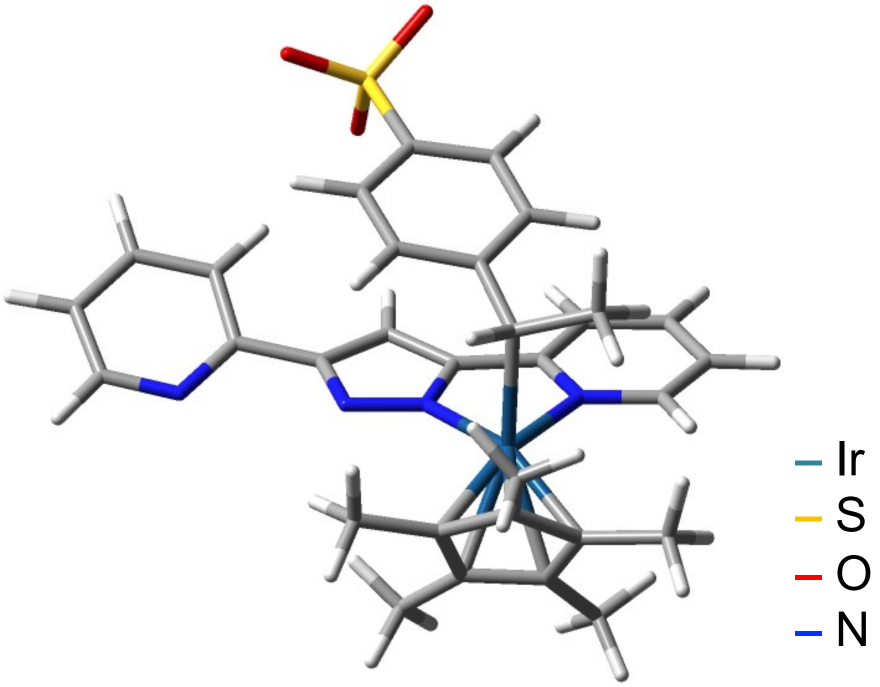

Fig. S12 A DFT-optimized structure of **Ir(EtBnS)-bpp** calculated at the B3LYP-D3BJ/LanL2TZ(f) (Ir), 6-31G* (Cu, H, C, O, N), 6-31+G* (S) level of theory.

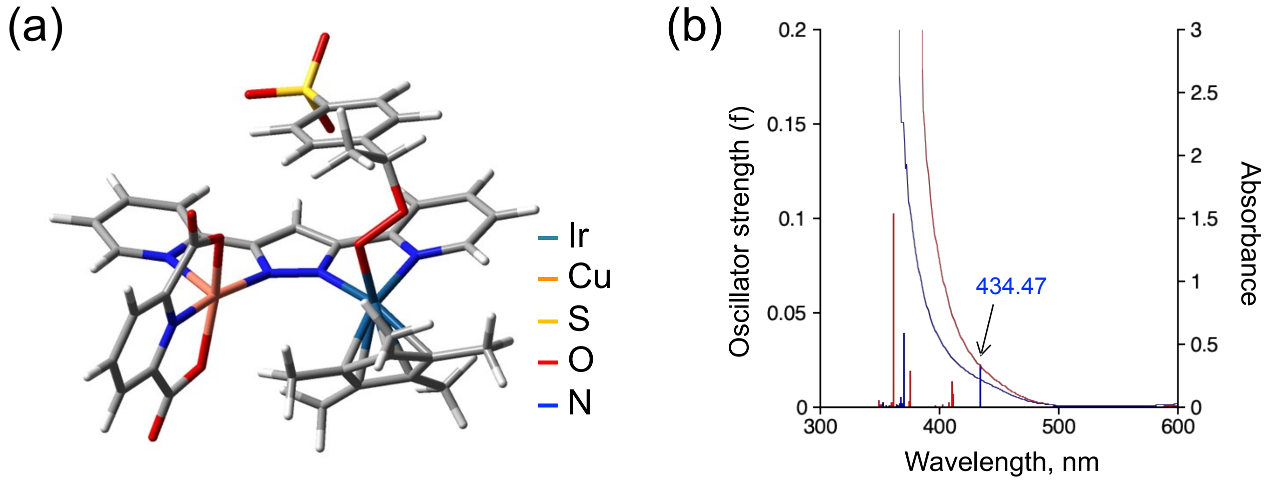

Fig. S13 (a) A DFT-optimized structure of **Ir(OOEtBnS)-Cu** as the reaction intermediate calculated at the B3LYP-D3BJ/LanL2TZ(f) (Ir), 6-31G* (Cu, H, C, O, N), 6-31+G* (S) level of theory. (b) TD-DFT calculations for 30 excited states of **Ir(OOEtBnS)-Cu** (blue) and **Ir(EtBnS)-Cu** (red) overlaid with UV-vis absorption spectra extracted from Fig. 2b at 9000 s under Ar (red) and 10500 s under O_2 (blue).

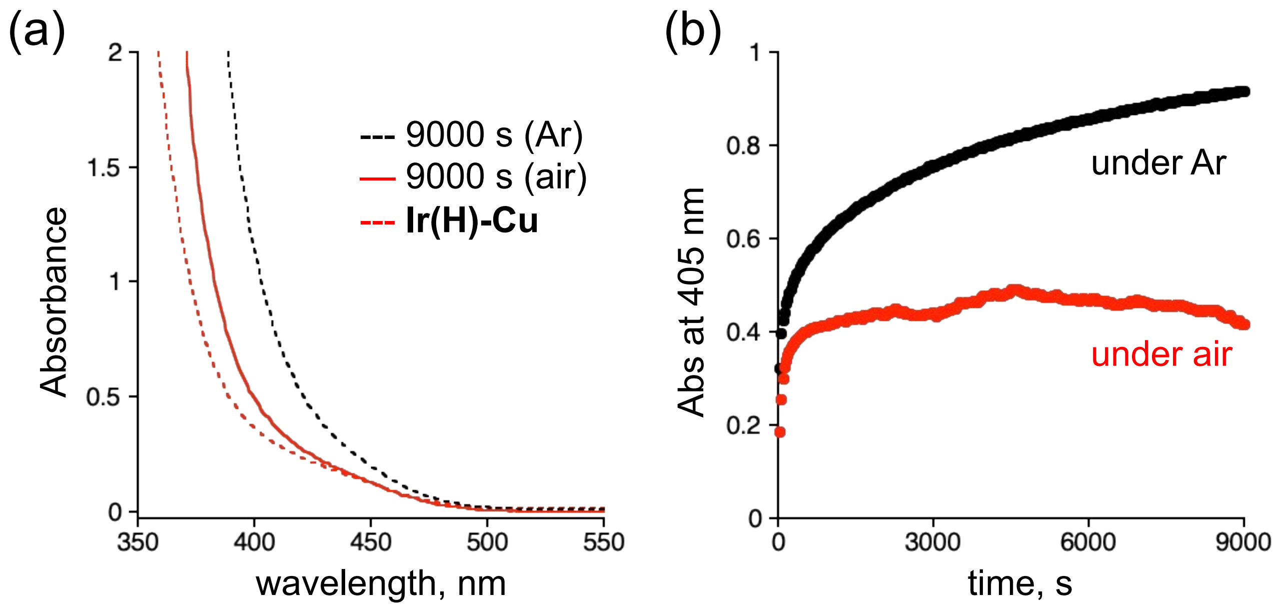

Fig. S14. (a) UV-Vis absorption spectral changes of **1** (0.10 mM) with **3** (5.0 mM) in formate buffer (0.60 M, [HCOOH]:[HCOONa] = 3:1) in the presence of 0.10 M StyreneS under Ar (black dashed line) and under air (red solid line) measured at 9000 s. The red dashed line denoted the Ir^{III}-H species (**Ir(H)-Cu**). (b) Time-profile of absorbance measured under Ar (black) and under air (red) monitored at 405 nm.

Table S1. Control experiments for the oxidation of StyreneS in water. [a]

entry	cat.	Yield, %	AcetophS
1	Ir-Cu (**1**)	10	112
2	Ir-Co (**5**)	n/a	none
3	Ir-Ni (**6**)	n/a	none

[a] Reaction conditions: [cat.] = 0.10 mM, [StyreneS] = 0.10 M, [HCOOH] = 0.20 M, solvent: D_2O, reaction temp.: 70 ºC., reaction time: 24 h.

Table S2. StyreneS oxidation with H_2O_2 as a terminal oxidant. [a]

cat.	additive	Yield, % (TON)		
		DiolS [d]	BenzaldehydeS [e]	AcetophS
Ir-Cu (**1**)	AcOH, AcONa [b]	27 (266)	12 (122)	none
none	AcOH, AcONa [b]	31 (–)	14 (–)	none
none	HCOOH, HCOONa [c]	19 (–)	7 (–)	none

[a] Reaction conditions: [cat.] = 0.10 mM, [StyreneS] = 0.10 M, [H_2O_2] = 0.10 M, solvent: D_2O, reaction temp.: 70 ºC., reaction time: 24 h. [b] Acetate buffer 0.60 M ([AcOH]:[AcONa] = 3:1). [c] Formate buffer 0.60 M ([HCOOH]:[HCOONa] = 3:1). [d] DiolS = 1,2-dihydroxyethylbenzene sulfonate [e] BenzaldehydeS = 4-formylbenzenesulfonate.

Table S3. Comparison of Ir(EtBnS)-Cu and Ir(EtBnS)-bpp electronic properties.

Complex	Mülliken charges				α-HOMO, a.u.	β-LUMO, a.u.
	Ir	Cu	$N_{pz\text{-}Ir}$	$N_{pz\text{-}Cu}$		
Ir(EtBnS)-Cu	+0.422	+0.801	–0.413	–0.430	–0.19396	–0.07949
Ir(EtBnS)-bpp	+0.410	–	–0.449	–0.319	–0.18434	–0.04703

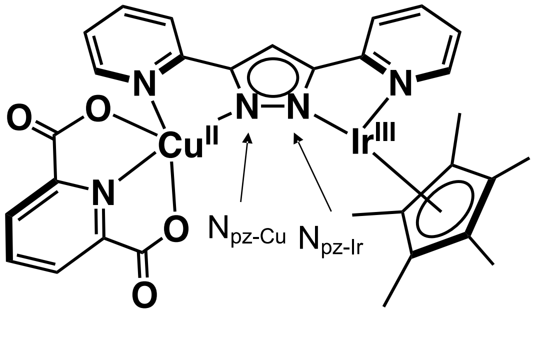

Table S4. Spin densities and Mülliken charges of DFT-optimized reaction intermediates.

Complex	Spin density		Mülliken charge			
	Ir	Cu	Ir	Cu	$N_{pz\text{-}Ir}$	$N_{pz\text{-}Cu}$
Ir(H)-Cu	-0.000094	0.709	+0.314	+0.798	–0.409	–0.426
Ir(EtBnS)-Cu	-0.000434	0.714	+0.422	+0.801	–0.413	–0.430
Ir(OOEtBnS)-Cu	-0.000439	0.710	+0.683	+0.782	–0.415	–0.433

Table S5. Cartesian coordinates of DFT-optimized **Ir(H)-Cu** at the unrestricted B3LYP-D3BJ/LanL2TZ(f) (Ir), 6-31G* (Cu, H, C, O, N) level of theory.

Atom	Coordinates (Angstroms) X	Y	Z
Cu	0.078100	-2.846600	2.966900
Ir	0.001300	0.008500	0.046200
H	1.592200	-0.101400	0.222200
O	1.982800	-2.965800	2.341800
O	3.595300	-4.490300	1.901200
O	-1.898900	-3.349500	3.110400
O	-3.219900	-5.171500	3.356000
N	0.199900	-4.736000	2.727400
C	2.443800	-4.171400	2.201300
C	1.391800	-5.248300	2.419900
C	1.540500	-6.624900	2.284300
H	2.507900	-7.041100	2.029500
C	0.413100	-7.432100	2.477700
H	0.498400	-8.509500	2.379400
C	-0.826900	-6.857800	2.780600
H	-1.719600	-7.456600	2.916700
C	-0.898300	-5.472700	2.895500
C	-2.134100	-4.623500	3.154200
N	0.230500	2.044400	0.511400
C	0.388900	3.016700	-0.408600
H	0.473500	2.689100	-1.434900
N	-0.026300	0.045400	2.104900
N	-0.064300	-0.908700	3.031700
N	0.153900	-2.493300	5.187700
C	0.453000	4.360100	-0.067800
H	0.575300	5.101900	-0.848300
C	0.364500	4.723600	1.279000
H	0.412400	5.765900	1.575400
C	0.222100	3.727200	2.236100
H	0.158600	3.964500	3.292000
C	0.157300	2.391800	1.830600
C	0.036700	1.259500	2.716800
C	0.031400	1.071300	4.104600
H	0.088000	1.825800	4.874500
C	-0.024200	-0.315400	4.261000
C	0.005000	-1.173800	5.443400
C	-0.105500	-0.686500	6.750300
H	-0.230500	0.375900	6.927600
C	-0.059600	-1.590100	7.808200
H	-0.143100	-1.235700	8.830600
C	0.088700	-2.952600	7.539600
H	0.126200	-3.685900	8.337100
C	0.189000	-3.354600	6.209400
H	0.302500	-4.402500	5.944900
C	-0.810100	-1.886300	-0.680900
C	-0.028400	-1.274200	-1.723800
C	-0.631200	-0.014900	-2.055000
C	-1.856000	0.113100	-1.266700
C	-1.964300	-1.025800	-0.431000
C	-0.642400	-3.282800	-0.169800

H	-1.100000	-4.001200	-0.862500
H	-1.128700	-3.402200	0.797900
H	0.411900	-3.545100	-0.058100
C	1.161600	-1.885300	-2.397200
H	1.712300	-2.537900	-1.715700
H	1.847400	-1.120400	-2.769300
H	0.831700	-2.489500	-3.251900
C	-0.230200	0.872400	-3.195300
H	-0.614100	0.468100	-4.140600
H	0.857000	0.951100	-3.284000
H	-0.640400	1.879800	-3.087900
C	-2.799500	1.272900	-1.347200
H	-2.260500	2.225400	-1.384400
H	-3.471900	1.300800	-0.486500
H	-3.412700	1.207600	-2.254400
C	-3.051600	-1.326600	0.553100
H	-3.801600	-1.985500	0.096600
H	-3.560500	-0.414900	0.876800
H	-2.661300	-1.837100	1.437600

Table S6. Cartesian coordinates of DFT-optimized **Ir(EtBnS)-Cu** at the unrestricted B3LYP-D3BJ/LanL2TZ(f) (Ir), 6-31G* (Cu, H, C, O, N), 6-31+G* (S) level of theory.

Atom	Coordinates (Angstroms) X	Y	Z
Cu	-1.057500	2.641000	3.121100
Ir	0.000300	0.052700	0.032400
C	-2.034100	-0.766200	0.107000
H	-2.652400	0.137100	0.076400
C	-2.364600	-1.497800	1.373600
C	-2.096500	-2.868500	1.546300
C	-2.932900	-0.808400	2.461300
C	-2.273900	-3.494400	2.778500
H	-1.700900	-3.450500	0.720400
C	-3.114700	-1.424300	3.694900
H	-3.182700	0.241500	2.348100
C	-2.752800	-2.763300	3.866200
H	-2.021100	-4.542100	2.903800
H	-3.510400	-0.865300	4.536300
C	-2.324100	-1.609400	-1.140600
H	-3.333800	-2.044600	-1.098500
H	-2.264600	-1.007400	-2.048600
H	-1.622000	-2.441100	-1.258500
S	-2.762500	-3.498000	5.501000
O	-3.834200	-2.792300	6.265100
O	-3.041200	-4.951200	5.293700
O	-1.395800	-3.256100	6.072700
O	-2.949600	2.435400	2.433400
O	-4.857400	3.618900	2.154300
O	0.757800	3.541000	3.375500
O	1.659900	5.569400	3.817300
N	-1.585900	4.481500	3.064200
C	-3.659900	3.520700	2.428100
C	-2.858200	4.762800	2.781200
C	-3.296600	6.083300	2.787000
H	-4.330200	6.309600	2.553300
C	-2.368000	7.085600	3.090400
H	-2.680300	8.125000	3.102000
C	-1.035600	6.756300	3.363600
H	-0.290500	7.511600	3.583400
C	-0.672000	5.413200	3.337500
C	0.715600	4.825700	3.542700
N	0.661300	-1.881300	0.434900
C	0.947500	-2.796400	-0.514400
H	0.785200	-2.485300	-1.536600
N	0.030300	-0.011900	2.094000
N	-0.419200	0.803300	3.047900
N	-1.299700	2.065000	5.264600
C	1.403400	-4.068700	-0.206500
H	1.616100	-4.766700	-1.007700
C	1.572700	-4.422600	1.137000
H	1.926000	-5.411800	1.407700
C	1.272900	-3.490500	2.119900
H	1.373800	-3.726100	3.173100
C	0.811600	-2.224600	1.747700

C	0.409700	-1.190000	2.664400
C	0.184100	-1.129800	4.042600
H	0.285700	-1.920400	4.769400
C	-0.348400	0.143500	4.242200
C	-0.919000	0.777400	5.425200
C	-1.121400	0.091800	6.629700
H	-0.869600	-0.960700	6.702200
C	-1.707700	0.774300	7.691600
H	-1.881000	0.265200	8.634800
C	-2.082000	2.111100	7.529800
H	-2.542000	2.672000	8.335600
C	-1.865400	2.709700	6.291200
H	-2.154600	3.740500	6.104300
C	0.050400	2.175300	-0.544900
C	-0.499600	1.425200	-1.634000
C	0.484000	0.469300	-2.070700
C	1.693800	0.707500	-1.288000
C	1.432400	1.746700	-0.356600
C	-0.583500	3.377500	0.079400
H	-0.493100	4.244900	-0.588100
H	-0.089500	3.631400	1.016900
H	-1.644000	3.214000	0.281500
C	-1.835400	1.694100	-2.252300
H	-2.620400	1.786400	-1.496100
H	-2.124900	0.913900	-2.957700
H	-1.793700	2.642600	-2.802000
C	0.401600	-0.393200	-3.294300
H	0.643200	0.192400	-4.190500
H	-0.596300	-0.816000	-3.430200
H	1.115100	-1.220700	-3.251200
C	2.986000	-0.026200	-1.469000
H	2.824500	-1.093700	-1.645700
H	3.628800	0.075100	-0.591500
H	3.529500	0.370000	-2.335700
C	2.387300	2.331200	0.637100
H	2.857700	3.234600	0.226700
H	3.184000	1.624600	0.885100
H	1.879900	2.614200	1.562900

Table S7. Cartesian coordinates of DFT-optimized **Ir(EtBnS)-bpp** at the B3LYP-D3BJ/LanL2TZ(f) (Ir), 6-31G* (Cu, H, C, O, N), 6-31+G* (S) level of theory.

Atom	Coordinates (Angstroms) X	Y	Z
Ir	-0.026600	-0.019100	-0.020800
N	0.673500	-1.944500	0.391300
C	1.014900	-2.848500	-0.549900
H	0.914100	-2.520900	-1.576200
N	-0.003700	-0.078100	2.013300
N	-0.368900	0.834300	2.909600
N	-0.655700	2.254100	5.398300
C	1.453500	-4.124500	-0.233400
H	1.715300	-4.812900	-1.028500
C	1.541000	-4.493800	1.115100
H	1.873500	-5.488300	1.394200
C	1.193500	-3.571600	2.090500
H	1.239600	-3.819700	3.144900
C	0.761100	-2.294000	1.713400
C	0.365300	-1.247800	2.614500
C	0.206300	-1.088500	3.992500
H	0.355400	-1.823500	4.768700
C	-0.256800	0.231500	4.130700
C	-0.677400	0.904200	5.364100
C	-1.114600	0.134000	6.459500
H	-1.164200	-0.947700	6.377000
C	-1.518600	0.783600	7.620700
H	-1.862700	0.209500	8.476800
C	-1.492200	2.179700	7.663000
H	-1.802400	2.728600	8.546200
C	-1.059700	2.857500	6.523500
H	-1.031700	3.946400	6.514700
C	-2.023500	-0.908100	-0.021200
H	-2.667600	-0.030800	-0.147200
C	-2.399800	-1.554900	1.274900
C	-2.202600	-2.924500	1.527100
C	-2.930200	-0.777700	2.321500
C	-2.447000	-3.475300	2.783700
H	-1.820200	-3.567900	0.741600
C	-3.165700	-1.310900	3.582100
H	-3.109300	0.279100	2.147200
C	-2.907200	-2.663800	3.821000
H	-2.268400	-4.530300	2.965200
H	-3.531800	-0.685600	4.388800
C	-2.214000	-1.830300	-1.230500
H	-3.225600	-2.264000	-1.248600
H	-2.071200	-1.285500	-2.166300
H	-1.507600	-2.666200	-1.236800
S	-3.057800	-3.331400	5.476600
O	-4.077100	-2.488600	6.170800
O	-3.486800	-4.752400	5.302100
O	-1.698100	-3.218800	6.099800
C	-0.184100	2.091500	-0.504900
C	-0.549900	1.351700	-1.683900
C	0.567000	0.540800	-2.079500

C	1.677200	0.860300	-1.185400
C	1.223300	1.814800	-0.233600
C	-1.018100	3.110400	0.210800
H	-0.774600	4.127000	-0.125300
H	-0.849700	3.052300	1.289500
H	-2.083400	2.943200	0.027700
C	-1.851800	1.484200	-2.409700
H	-2.701400	1.519200	-1.722300
H	-2.012600	0.664200	-3.111700
H	-1.852800	2.419400	-2.983500
C	0.666800	-0.293000	-3.321300
H	0.950300	0.323600	-4.184300
H	-0.282100	-0.781600	-3.557300
H	1.428000	-1.071800	-3.218000
C	3.049100	0.268100	-1.289800
H	3.004600	-0.807400	-1.489700
H	3.617800	0.413100	-0.368100
H	3.609900	0.731700	-2.111200
C	1.997100	2.432500	0.889100
H	2.227900	3.481800	0.666100
H	2.940600	1.908700	1.062800
H	1.413800	2.402000	1.814900

Table S8. Cartesian coordinates of DFT-Optimized **Ir(OOEtBnS)-Cu** at the unrestricted B3LYP-D3BJ/LanL2TZ(f) (Ir), 6-31G* (Cu, H, C, O, N), 6-31+G* (S) level of theory.

Atom	Coordinates (Angstroms)		
	X	Y	Z
Cu	-1.512600	2.601400	1.222800
Ir	0.684600	-0.633200	-0.240400
O	-1.235700	-1.303800	-0.304900
O	-1.290600	-2.770400	-0.387900
C	-2.425100	-3.196100	0.376000
H	-2.396300	-4.281800	0.213200
C	-3.732900	-2.651500	-0.198500
H	-4.589500	-3.077800	0.335000
H	-3.782000	-1.563500	-0.122100
H	-3.809700	-2.926000	-1.255700
C	-2.233400	-2.944500	1.861300
C	-1.583600	-3.916500	2.632300
C	-2.655400	-1.763800	2.485100
C	-1.332200	-3.712200	3.986500
H	-1.251200	-4.837500	2.160400
C	-2.412000	-1.551200	3.841900
H	-3.154500	-0.990300	1.914000
C	-1.739700	-2.519500	4.587600
H	-0.811500	-4.465900	4.567700
H	-2.715100	-0.628400	4.321800
S	-1.305900	-2.212000	6.304200
O	-2.006000	-0.953200	6.692700
O	-1.772100	-3.414500	7.056400
O	0.187600	-2.070700	6.322400
O	-3.183500	1.524400	0.907800
O	-5.170300	1.609400	-0.167400
O	-0.088400	4.036500	0.978300
O	0.184800	6.115400	0.127600
N	-2.459400	3.815500	0.085900
C	-4.082200	2.086100	0.161600
C	-3.678800	3.467600	-0.327200
C	-4.410500	4.329700	-1.137900
H	-5.398100	4.041600	-1.477800
C	-3.832200	5.554400	-1.490900
H	-4.379700	6.248000	-2.120900
C	-2.548200	5.885700	-1.043400
H	-2.071600	6.822000	-1.307800
C	-1.875400	4.968900	-0.241400
C	-0.470200	5.090300	0.325300
N	1.235100	-2.185500	1.065800
C	1.637000	-3.405200	0.666000
H	1.586800	-3.599000	-0.395600
N	0.220600	0.115500	1.626200
N	-0.417900	1.204200	2.057800
N	-1.792700	3.114500	3.375500
C	2.085900	-4.364700	1.562800
H	2.410300	-5.330300	1.192300
C	2.100500	-4.062700	2.927700
H	2.439100	-4.797200	3.651100
C	1.643300	-2.821800	3.352100

H	1.563100	-2.568800	4.403500
C	1.210600	-1.898700	2.397100
C	0.642500	-0.607700	2.697900
C	0.279700	0.054500	3.872400
H	0.419100	-0.296600	4.883600
C	-0.408100	1.183800	3.424000
C	-1.155900	2.206500	4.148800
C	-1.248300	2.235100	5.545700
H	-0.766100	1.466700	6.138100
C	-2.000500	3.241700	6.143700
H	-2.089900	3.284100	7.224700
C	-2.641300	4.189900	5.342200
H	-3.234600	4.988100	5.773900
C	-2.511700	4.078800	3.959900
H	-2.999600	4.780800	3.288900
C	0.734900	0.972500	-1.698500
C	0.678800	-0.276200	-2.416100
C	1.824600	-1.058400	-2.066100
C	2.644800	-0.259000	-1.156300
C	1.984200	0.988400	-0.944900
C	-0.211600	2.117400	-1.871500
H	0.098900	2.757600	-2.707500
H	-0.238700	2.731400	-0.973000
H	-1.226200	1.764100	-2.075100
C	-0.453400	-0.689500	-3.300000
H	-1.390400	-0.664700	-2.734100
H	-0.312700	-1.702700	-3.682300
H	-0.538400	-0.006100	-4.152100
C	2.215800	-2.373900	-2.667500
H	2.701800	-2.214400	-3.638100
H	1.348500	-3.019700	-2.829900
H	2.927400	-2.908800	-2.033900
C	3.966300	-0.683000	-0.594100
H	3.962800	-1.743600	-0.325600
H	4.217800	-0.110200	0.301600
H	4.763500	-0.528800	-1.331600
C	2.477200	2.128900	-0.111500
H	3.060100	2.818100	-0.736000
H	3.125300	1.778100	0.695800
H	1.650800	2.692700	0.327400

Table S9. Cartesian coordinates of DFT-Optimized **Ir(H)-Cu|||StyreneS** at the unrestricted B3LYP-D3BJ/LanL2TZ(f) (Ir), 6-31G* (Cu, H, C, O, N), 6-31+G* (S) level of theory.

Atom	Coordinates (Angstroms) X	Y	Z
Cu	0.117400	-2.869900	3.117300
Ir	0.157500	-0.076300	0.146600
H	1.710900	-0.291300	0.393200
O	2.069600	-2.927900	2.637400
O	3.743500	-4.391200	2.224900
O	-1.850400	-3.439900	3.128700
O	-3.126100	-5.307100	3.232200
N	0.312300	-4.751500	2.839700
C	2.568600	-4.113400	2.474000
C	1.534900	-5.222500	2.590300
C	1.730700	-6.588800	2.415500
H	2.723700	-6.968900	2.207000
C	0.617500	-7.432300	2.508500
H	0.739300	-8.502700	2.377600
C	-0.654300	-6.901500	2.752700
H	-1.536600	-7.527700	2.810700
C	-0.772100	-5.523800	2.911000
C	-2.047400	-4.720400	3.117600
N	0.531700	1.939900	0.590400
C	0.842500	2.874600	-0.328300
H	0.965300	2.521300	-1.341100
N	0.061600	-0.001500	2.196000
N	-0.073500	-0.935900	3.135000
N	0.047200	-2.486900	5.321900
C	1.009600	4.212800	-0.001400
H	1.252600	4.924400	-0.782000
C	0.865700	4.610100	1.330900
H	0.990300	5.649500	1.615600
C	0.580400	3.648000	2.290100
H	0.488600	3.904200	3.339200
C	0.425600	2.316200	1.899100
C	0.181300	1.215000	2.793700
C	0.109100	1.052800	4.182200
H	0.254100	1.801000	4.945000
C	-0.040700	-0.323400	4.356100
C	-0.097500	-1.162400	5.548700
C	-0.287900	-0.656000	6.839300
H	-0.405400	0.410900	6.990700
C	-0.315900	-1.542700	7.910100
H	-0.458600	-1.172400	8.920600
C	-0.163500	-2.911600	7.671500
H	-0.181000	-3.632800	8.480900
C	0.011600	-3.334000	6.356600
H	0.129000	-4.386800	6.113500
C	-0.665600	-1.944400	-0.617200
C	0.151500	-1.333500	-1.637100
C	-0.428100	-0.065800	-1.971200
C	-1.675700	0.071300	-1.220200
C	-1.817300	-1.072500	-0.397300
C	-0.525100	-3.344100	-0.104900

H	-1.005600	-4.057300	-0.788000
H	-0.999800	-3.448700	0.870300
H	0.524900	-3.629700	-0.004000
C	1.355300	-1.950200	-2.280900
H	1.893100	-2.595000	-1.581400
H	2.048700	-1.187500	-2.643200
H	1.047700	-2.562800	-3.138000
C	0.027800	0.837200	-3.077900
H	-0.340000	0.469200	-4.044200
H	1.119300	0.885800	-3.133600
H	-0.355000	1.853400	-2.952700
C	-2.603400	1.243000	-1.320200
H	-2.050000	2.187500	-1.349100
H	-3.289300	1.282000	-0.470300
H	-3.204400	1.186800	-2.236600
C	-2.930300	-1.368400	0.559700
H	-3.670300	-2.027700	0.087500
H	-3.445500	-0.454900	0.868700
H	-2.561500	-1.877400	1.454400
C	4.039200	1.895600	-0.277000
C	3.900100	1.646000	1.164000
C	3.814500	0.353100	1.711000
C	3.825700	2.740400	2.042200
C	3.643200	0.158700	3.077000
H	3.880400	-0.513600	1.063100
C	3.656900	2.556800	3.411800
H	3.877800	3.747800	1.639000
C	3.562900	1.263000	3.928300
H	3.542100	-0.844000	3.474600
H	3.575800	3.411300	4.075300
C	3.969700	0.987300	-1.260500
S	3.466000	1.026900	5.707200
O	2.523100	2.073000	6.206600
O	2.989300	-0.369100	5.920200
O	4.865500	1.240800	6.196200
H	4.083900	1.281000	-2.299600
H	3.789200	-0.066400	-1.071100
H	4.197900	2.939500	-0.545200
